# Assessing residential activity in a home plumbing system simulator: monitoring the occurrence and relationship of major opportunistic pathogens and phagocytic amoebas

**DOI:** 10.3389/fmicb.2023.1260460

**Published:** 2023-10-17

**Authors:** Vicente Gomez-Alvarez, Hodon Ryu, Min Tang, Morgan McNeely, Christy Muhlen, Megan Urbanic, Daniel Williams, Darren Lytle, Laura Boczek

**Affiliations:** ^1^Office of Research and Development, U.S. Environmental Protection Agency, Cincinnati, OH, United States; ^2^Oak Ridge for Science and Education Research Fellow at U.S. Environmental Protection Agency, Cincinnati, OH, United States

**Keywords:** *Legionella pneumophila*, premise plumbing, microbial communities, opportunistic pathogens, free-living amoeba

## Abstract

Opportunistic premise plumbing pathogens (OPPPs) have been detected in buildings’ plumbing systems causing waterborne disease outbreaks in the United States. In this study, we monitored the occurrence of OPPPs along with free-living amoeba (FLA) and investigated the effects of residential activities in a simulated home plumbing system (HPS). Water samples were collected from various locations in the HPS and analyzed for three major OPPPs: *Legionella pneumophila*, nontuberculous mycobacterial species (e.g., *Mycobacterium avium*, *M. intracellulare*, and *M. abscessus*), and *Pseudomonas aeruginosa* along with two groups of amoebas (*Acanthamoeba* and *Vermamoeba vermiformis*). A metagenomic approach was also used to further characterize the microbial communities. Results show that the microbial community is highly diverse with evidence of spatial and temporal structuring influenced by environmental conditions. *L. pneumophila* was the most prevalent pathogen (86% of samples), followed by *M. intracellulare* (66%) and *P. aeruginosa* (21%). Interestingly, *M. avium* and *M. abscessus* were not detected in any samples. The data revealed a relatively low prevalence of *Acanthamoeba* spp. (4%), while *V. vermiformis* was widely detected (81%) across all the sampling locations within the HPS. Locations with a high concentration of *L. pneumophila* and *M. intracellulare* coincided with the highest detection of *V. vermiformis*, suggesting the potential growth of both populations within FLA and additional protection in drinking water. After a period of stagnation lasting at least 2-weeks, the concentrations of OPPPs and amoeba immediately increased and then decreased gradually back to the baseline. Furthermore, monitoring the microbial population after drainage of the hot water tank and partial drainage of the entire HPS demonstrated no significant mitigation of the selected OPPPs. This study demonstrates that these organisms can adjust to their environment during such events and may survive in biofilms and/or grow within FLA, protecting them from stressors in the supplied water.

## Graphical abstract

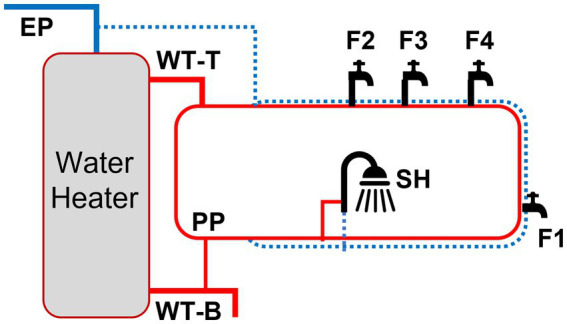

## Introduction

The function of a drinking water distribution system is to deliver potable water to all customers of the system in sufficient quantity, at the appropriate pressure, with minimal loss, and with acceptable quality (National Research Council, 2006). Following collection and appropriate treatment, drinking water is supplied to end users *via* a distribution system that includes service connection lines. Premise plumbing includes that portion of the drinking water distribution system connected *via* the service line to public and private houses and other buildings (National Research Council, 2006). Water quality within the premise plumbing is not monitored by the United States Environmental Protection Agency (US EPA) regulations, except for the Lead and Copper Rule (LCR). Previously identified problems (including microbial growth) in the drinking water distribution system can also occur in premise plumbing. Studies in homes and occupational buildings have suggested that the water quality within premise plumbing is influenced by a range of factors that are associated with the delivered water quality involving water treatment changes such as a switch from chlorine to chloramine (Pryor et al., 2004), seasonality (Zlatanović et al., 2017), and pressure zone (Haig et al., 2020). In addition, microbial proliferation and growth within plumbing are associated with the deterioration of physico-chemical water quality due to degradation and corrosion of pipe materials (Cullom et al., 2020). As a result, premise plumbing conditions can amplify the potential public health risk relative to the drinking water distribution system. The unique characteristics of premise plumbing (e.g., large surface area to volume ratio, long water age, favorable temperature, and low or no disinfectant residual) provide a favorable environment for microbial proliferation and growth within plumbing surface biofilms, including opportunistic premise plumbing pathogens (OPPPs) that cause infectious disease (National Research Council, 2006). These built environments provide unique conditions for OPPPs exposure *via* aerosolized water droplets produced by showerheads, faucets, and tubs (Logan-Jackson et al., 2023).

Public health data show a significant fraction of the nation’s waterborne disease outbreaks is attributable to premise plumbing in houses and occupational buildings. Surveillance data in the US indicate an increasing trend in the annual proportion of reported outbreaks associated with premise plumbing deficiencies (Craun et al., 2010; Collier et al., 2021). The most recent drinking water-associated outbreak surveillance data indicates that drinking water exposure was associated with 28% of outbreaks, resulting in 34% of reported cases with nearly 70% of reported hospitalizations, and 93% of deaths (Centers for Disease Control and Prevention, 2022). The 2015 is the latest year a summary report is available due to limited capacity for waterborne disease surveillance and competing response priorities at the Centers for Disease Control and Prevention (CDC). Comprehensive assessments of the health burden of infectious disease in the United States in 2014 estimated 6,630 deaths attributed to waterborne transmission, with most hospitalizations and deaths caused by these opportunistic pathogens, costing $2.39 billion annually (Collier et al., 2021).

Pathogenic bacteria previously recognized as biofilm-associated OPPPs include *Legionella pneumophila*, nontuberculous mycobacterial (NTM) species, and *Pseudomonas aeruginosa* (Falkinham et al., 2015). *L. pneumophila* is the primary causative agent of Legionnaires’ disease (Phin et al., 2014), in which serogroup 1 has been implicated in most cases associated with built environments (Cunha et al., 2016). Despite the implementation of disinfection strategies to mitigate their presence, this Gram-negative bacterium can colonize and persist within building water systems (Berjeaud et al., 2016). Legionnaires’ disease occurs sporadically and in outbreaks, with the sporadic form representing up to 82% of the cases (Sopena et al., 2007). Nevertheless, reported cases of Legionnaires’ disease in the United States have grown nine-fold since 2000 (Centers for Disease Control and Prevention, 2020). NTM species (e.g., *Mycobacterium avium*, *M. intracellulare*, and *M. abscessus*) are increasingly recognized as important opportunistic pathogens of humans (Dahl et al., 2022). In the United States, most pulmonary NTM infections are caused by *M. avium-intracellulare* complex, with other species, such as *M. abscessus* complex, also contributing to this disease (Winthrop et al., 2020). Data indicates a global increase in NTM for both *Mycobacterium* complexes in reports of infection and disease (Dahl et al., 2022). *P. aeruginosa* is widely occurring in premise plumbing and is recognized for its capacity to form or join biofilms in point-of-use devices, such as faucets, drains, sinks, and showerheads (Bédard et al., 2016). This opportunistic pathogen is an emerging worldwide public health threat and one of the leading causes of nosocomial infections (Moradali et al., 2017).

**Figure 1:**
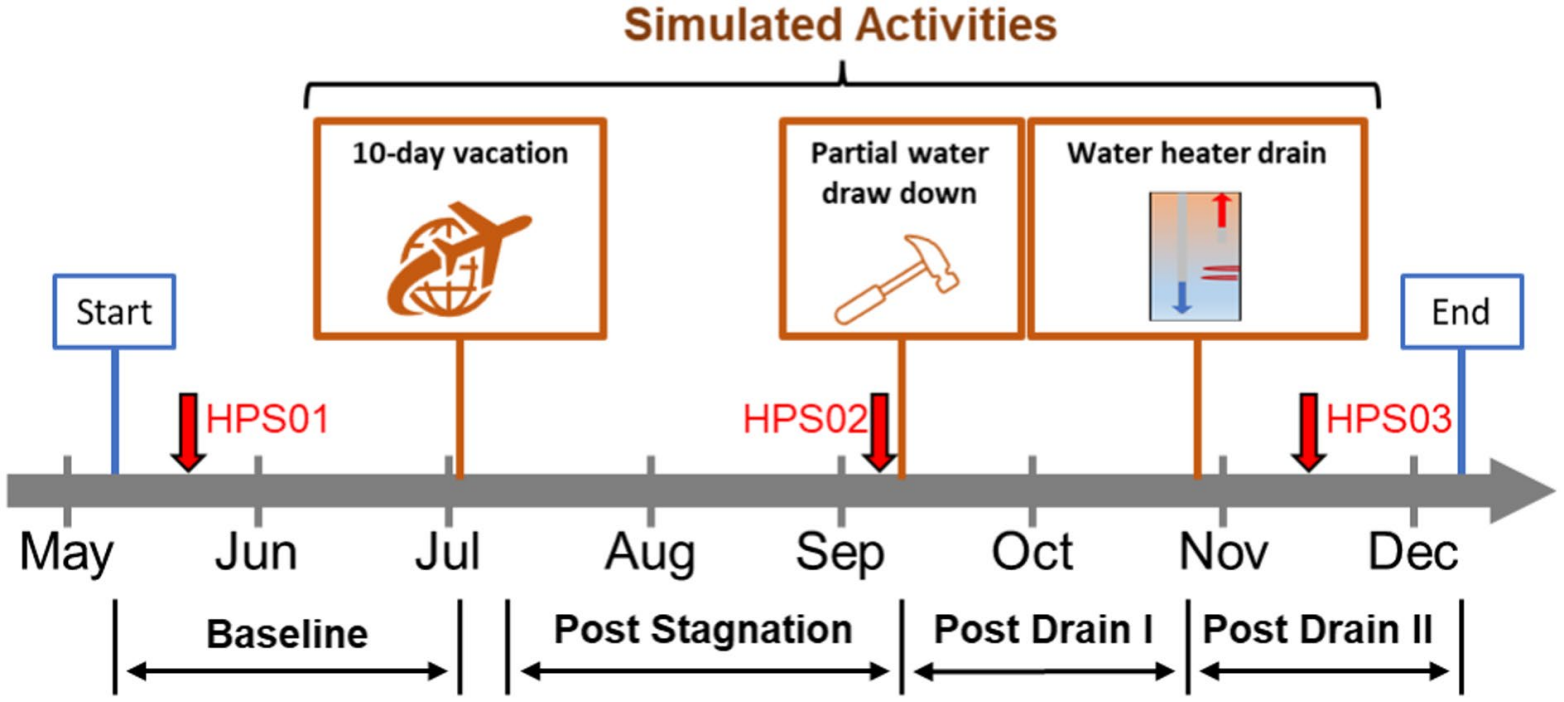
Model home simulated activities. Timeline of simulated residential activities performed during the study. Red arrows represent sampling events for metagenomics analysis. Orange boxes represent residential activities: stagnation (i.e., 10-day vacation), home repair, and water heater maintenance. Flushing events: baseline (BL); post stagnation (PS); post drain I (PD I); post drain II (PD II).

Previous studies examined the physico-chemical and microbial water quality changes in buildings and their associations (Lautenschlager et al., 2010; Zlatanović et al., 2017; Salehi et al., 2018; Ley et al., 2020; Rhoads et al., 2020). To better understand premise plumbing issues, a home plumbing system (HPS) simulator was constructed in the EPA’s Andrew W. Breidenbach Environmental Research Center (AWBERC) in Cincinnati, Ohio (Lytle et al., 2021). There have already been early studies using the same HPS about the seasonal variation and effect of temperature on OPPPs (Lu et al., 2017a) and the bacterial community diversity (Zhang et al., 2021). However, when residential homes are already contaminated with *Legionella* (and other OPPPs), no data are available to elucidate how residential activities such as plumbing and heater draining due to residential repair and maintenance would influence the drinking water quality. Therefore, the objective in this study was to characterize the microbial community and examine how populations of OPPPs along with free-living amoeba (FLA) responded to residential activities. Specifically, water samples were collected from various locations in the same HPS during simulated residential plumbing activities (Figure 1) and analyzed for three major OPPPs (*L. pneumophila*, NTM species [e.g., *M. avium*, *M. intracellulare*, and *M. abscessus*], and *P. aeruginosa*) and two groups of amoebas (*Acanthamoeba* and *Vermamoeba vermiformis*) using quantitative polymerase chain reaction (qPCR). A metagenomic approach was also used to characterize the microbial communities further. We hypothesized that residential activities (i.e., episodes of disturbances) induce a selection pressure on the bacterial community and the OPPP population. The outcome of this study will shed light on the role of residential plumbing activities on water quality and may indicate the mechanisms of these organisms to adjust to their environment during such events, protecting them from stressors in the supplied water.

## Materials and methods

### Home plumbing system simulator

The Home plumbing system (HPS) simulator was constructed in the EPA’s AWBERC (Cincinnati, Ohio) and has been continuously operated since 2012. Details of the layout and dimensions of the HPS were described previously (Lytle et al., 2021). The simulated HPS includes copper pipes, a 40-gallon electric hot water tank with a recirculating hot water pump, a dishwasher and washing machine, bathroom sinks, a shower, and a toilet (Supplementary Figure S1). The cold water lines and the hot water tank are fed with water that is supplied by the building water supply (Greater Cincinnati Water Works tap water). In May 2021, a new “Random Reduced Normal Use” flushing protocol was started to establish the baseline (BL) water quality for this study. The flush protocol (i.e., daily use by a four-person residence) consisted of opening and running the faucets, shower, and toilet for a combined volume of approximately 70–80 gallons per day. Water temperature and pH were measured with a handheld thermometer (Fisher Scientific, Hampton, NH) and a pH electrode (HACH, Loveland, CO), respectively. Free and total chlorine residuals were measured using the N, N-dimethyl-p-phenylenediamine (DPD) colorimetric method 4,500-CL G (Lipps et al., 2023). Detailed information on the design and flushing protocol for the HPS can be found in Supplementary materials and methods.

### Residential activity simulations

Three residential activities were simulated between May–December 2021: (i) extended stagnation (i.e., no use of water during a 10-day vacation), (ii) home plumbing repair, and (iii) water heater maintenance (Figure 1). The first activity simulating a resident’s long vacation was completed by fully stagnating the HPS for 10 days (i.e., no water use). Then the flush protocol (post stagnation: PS) was resumed, and the second activity simulating partial drawdown during home repair and maintenance was conducted by fully draining both hot and cold-water lines but not the water heater for 2 h. Once the partial drawdown was completed, the flush protocol (post drain I: PD I) was resumed. Finally, the third activity, simulating maintenance of the water heater as recommended by the manufacturer (i.e., cleaning), was executed by fully draining the water heater only. After completing the third activity, the flush protocol (post drain II: PD II) was resumed and continued until the end of the study.

### Sampling locations and collection

Samples were obtained from nine locations in the HPS: cold-water entry point (EP), hot water tank point of entry and return (WT-T and WT-B, respectively), hot water premise plumbing recirculation (PP), faucets (F1, F2, F3, and F4), and the shower (SH) (Supplementary Figure S1). Sample locations were assigned into three sections: (i) entry point (EP), (ii) water tank and recirculation (WT: WT-T, WT-B, and PP), and (iii) point of use (POU: F1, F2, F3, F4, and SH). Hot water samples (1 liter) were obtained from WT, PP and POU following 63 h of stagnation each week across the simulated residential activities unless stated otherwise, along with EP cold water samples as a comparison. We chose direct water sampling of household plumbing for qPCR instead of biofilm sampling to enhance our sampling effort and co-analyze with physico-chemical water parameters. For metagenomic analysis, bulk water (EP, WT-T, WT-B, and PP) and biofilms (F1–F4) were collected from three flushing events: BL, PS, and PD II (Figure 1). Briefly, 2 L water samples were filtered through a 0.2 μm sterile membrane filter (Pall Corporation, Port Washington, NY) and then cut aseptically to increase surface area for DNA extraction. Biofilms in faucets were harvested by scraping the inner surface of the pipes using sterile swabs.

### Quantitative polymerase chain reaction analyses

DNA of each water sample (approximately 850 ml) was immediately extracted *via* filtration using 0.2 μm sterile membrane (Pall Corporation, Port Washington, NY) according to the manufacturer’s protocol (DNeasy PowerWater kit, Qiagen). DNA concentration was measured using a NanoDrop ND-1000 UV spectrophotometer (NanoDrop Technologies, Wilmington, DE). DNA extracts were stored at −20°C until further processing.

Culture-independent qPCR analyses were conducted for three major OPPP groups: *Legionella pneumophila*, nontuberculous mycobacterial species (e.g., *Mycobacterium avium*, *M. intracellulare*, and *M. abscessus*), and *Pseudomonas aeruginosa* and two amoebas (*Acanthamoeba* and *Vermamoeba vermiformis*) (Supplementary Table S1). All qPCR assays were performed using a QuantStudio™ 6 Flex system (Applied Biosystems, Foster City, CA) following the procedure described in Ryu et al. (2013). Detailed information on probes, gene targets, and amplification protocols from the purified DNA can be found in Supplementary materials and methods and Supplementary Table S1.

### DNA extraction, sequencing, and processing of metagenomic libraries

Total DNA was extracted using the PureFood GMO and Authentication cartridge-based purification kit with the Maxwell® RSC 48 Instrument (Promega, Madison, WI) following the manufacturer’s instructions. Briefly, Filters and swabs were resuspended with 200 μl of CTAB (Promega, Part# MC1411) and 30 μl of Proteinase K (Promega, Part# MC500C), vortexed to mix for 30 s, and incubated at 70°C/5 min in a heat block. Then, 300 μl of Lysis Buffer (Promega, Part# A826D) was added, vortexed to mix for 10 s, and centrifuged. The supernatant was transferred (~1.0 ml) into the top reservoir in the Maxwell cartridge, and 80 μl of elution buffer was added into each collection tube. DNA concentrations were measured using the Quantus™ Fluorometer (Promega, Madison, WI) and stored at −80°C.

Paired-end standard metagenome libraries were prepared using the Illumina HiSeq in the NovaSeq 6,000 S2 PE150 XP sequence mode. Libraries were quality-checked using a 2,100 Bioanalyzer Instrument (Agilent, Santa Clara, USA). Prior to analysis, the 150-nucleotide (nt) pair-end reads were subjected to quality filtering and cleaning from adapters and phiX artifacts, error corrected, normalized (≤100×), and filtered to a minimum length of 100-nt using the bioinformatics software package BBMap v38.22^1^ with the following parameters: ktrim = r, k = 23, mink = 11, hdist = 1, tbo, tpe, maxns = 0, trimq = 10, qtrim = r, maq = 12, minlength = 100, ecco = t, eccc = t, ecct = t, and target = 100. The libraries contained an average ± standard error (±SE) of 29,397,634 ± 6,670,930 reads per sample.

### Taxonomic and diversity analysis of metagenomes

Taxonomy was assigned using Kraken2 v2.1.2 (Wood et al., 2019) with a confidence value of 0.1 for a taxonomic assignment using the pre-built custom Genome Taxonomy Database (GTDB) release 207^2^. Taxonomy counts for each sample were summarized by collapsing taxonomic assignments to the phylum, class, order, family, and genus level with Bracken v.2.8 (Lu et al., 2017b). A Sankey diagram was created using the online tool SankeyMATIC^3^ to illustrate the taxonomic flow lineages. Diversity indices were calculated based on the rarefied species abundance table across each sample generated by Bracken.

### Multivariable ordination and statistical analysis

Principal Coordinated Analysis (PCoA) was used to describe the relationships among microbial communities and was based on the Bray–Curtis similarity matrix. A one-way permutational multivariate analysis of variance (PERMANOVA) test was applied to the distance matrix with 9,999 permutations to determine if there were significant differences (*α* = 0.05) between the microbial communities (Anderson, 2001). The non-parametric Kendall’s tau correlation was calculated for each pairwise combination of variables (e.g., OPPP abundance, temperature, pH, and free chlorine) in the samples analyzed. A conventional approach was used to interpret the correlation coefficient (Schober et al., 2018) as follows: 0.00–0.09, negligible; 0.10–0.39, weak; 0.40–0.69, moderate, 0.70–0.89, strong; 0.90–1.00, very strong. The significance of the correlation test was set at 95% (*p* ≤ 0.05). The non-parametric Kruskal-Wallis one-way analysis of variance test for equal medians (*α* = 0.05) was used to evaluate the differences in diversity indices and water quality parameters between HPS sections and flushing events. Ordination plots and statistical analysis were performed with the software PAST v4.12b (Hammer et al., 2001). The gene copy number (GCN) generated from qPCR assays was transformed into log_10_ (*x* + 1) GCN.

## Results

### Water quality in HPS sections and flushing events after residential activities

**Figure 2:**
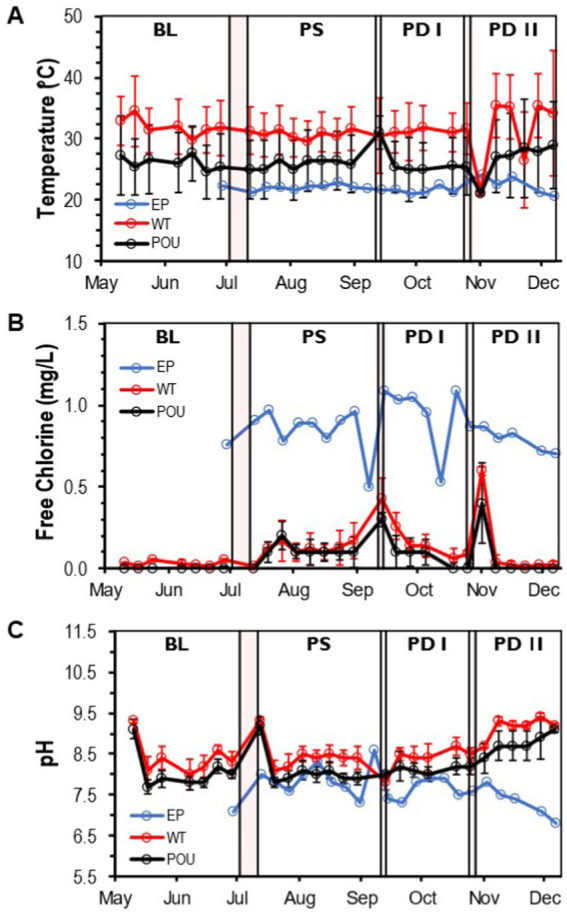
Water quality parameters in the HPS. Weekly concentration and measurements of **(A)** temperature, **(B)** free chlorine residual, and **(C)** pH at each section from May to December. Clear and red boxes represent flushing events and simulated residential activities, respectively (see Figure 1). Sections: entry point (EP); water tank and recirculation (WT); point-of-use (POU). Flushing events: baseline (BL); post stagnation (PS); post drain I (PD I); post drain II (PD II). Bars represent ± standard deviation (±SD).

A total of 211 water samples were collected from May to December 2021 and analyzed from three sections (entry point: 22 samples, water tank and recirculation: 54, point-of-use: 134) in the HPS. Water temperature, free chlorine residual, and pH were measured to reflect the quality of the entry water (EP) and hot water (WT and POU) sections (Supplementary Figure S1). A flush protocol (i.e., daily use by a four-person residence) was started in the HPS to establish the baseline (BL) water quality and resumed after completing each of the three simulated residential activities (PS, PD I, and PD II). EP samples (i.e., cold water) had an average water temperature of 22.1°C (±0.9 SD), free chlorine residual of 0.86 mg/L (±0.16 SD), and a pH of 7.6 (±0.4 SD) (Supplementary Figure S2). No significant differences were observed between the sample medians of measured water quality parameters in the EP water when using aggregate monthly data (Kruskal–Wallis, Dunn’s test *p* > 0.05, Supplementary Table S2). Throughout the study, the measured water quality parameters for the EP water remained stable and relatively constant (Figure 2).

Compared to cold water samples, the Kruskal–Wallis one-way analysis of variance (ANOVA) showed that all physico-chemical parameters within hot water samples were significantly different from the EP water (Supplementary Figure S2). The temperature increased to an average of 31.3°C (±4.3 SD) in the WT section and decreased significantly to 25.5°C (±4.9 SD) in the POU (Kruskal–Wallis, *H* = 50.6, Dunn’s test *p* < 0.0001; Supplementary Table S2 and Supplementary Figure S2A). Chlorine residuals were almost entirely depleted in hot water locations and were significantly different from the EP (Kruskal–Wallis, *H* = 62.1, Dunn’s test *p* < 0.0001). The average free chlorine value for the WT is 0.11 mg/L (±0.14 SD) and 0.07 mg/L (±0.10 SD) for the POU section (Supplementary Table S2 and Supplementary Figure S2B). The pH in hot water samples increased significantly to 8.6 (±0.5 SD) in WT and 8.2 (±0.5 SD) in POU samples compared to EP samples (Kruskal–Wallis, *H* = 52.9, Dunn’s test *p* < 0.0001; Supplementary Table S2 and Supplementary Figure S2C). Chlorine and pH followed similar tendencies across the study, while some variation was observed with temperature in the WT and POU sections (Figure 2).

No significant difference in hot water temperature was observed during flushing events between simulated residential activity in WT (Kruskal–Wallis, *H* = 1.3, Dunn’s test *p* > 0.05) and POU (Kruskal–Wallis, *H* = 7.6, Dunn’s test *p* > 0.05) sections (Supplementary Figure S3). In contrast, significant differences in the sample medians of free chlorine were observed only after stagnation (i.e., 10-day vacation) and water heater maintenance for both WT and POU (Kruskal–Wallis, Dunn’s test *p* < 0.001) sections (Supplementary Figure S4). An increase in pH (Kruskal–Wallis, Dunn’s test *p* < 0.001) was noticed after the water heater maintenance for both hot water sections (Supplementary Figure S5). The home repair residential activity appears to have no effect on the observed physico-chemical parameters.

### Microbial communities

**Figure 3:**
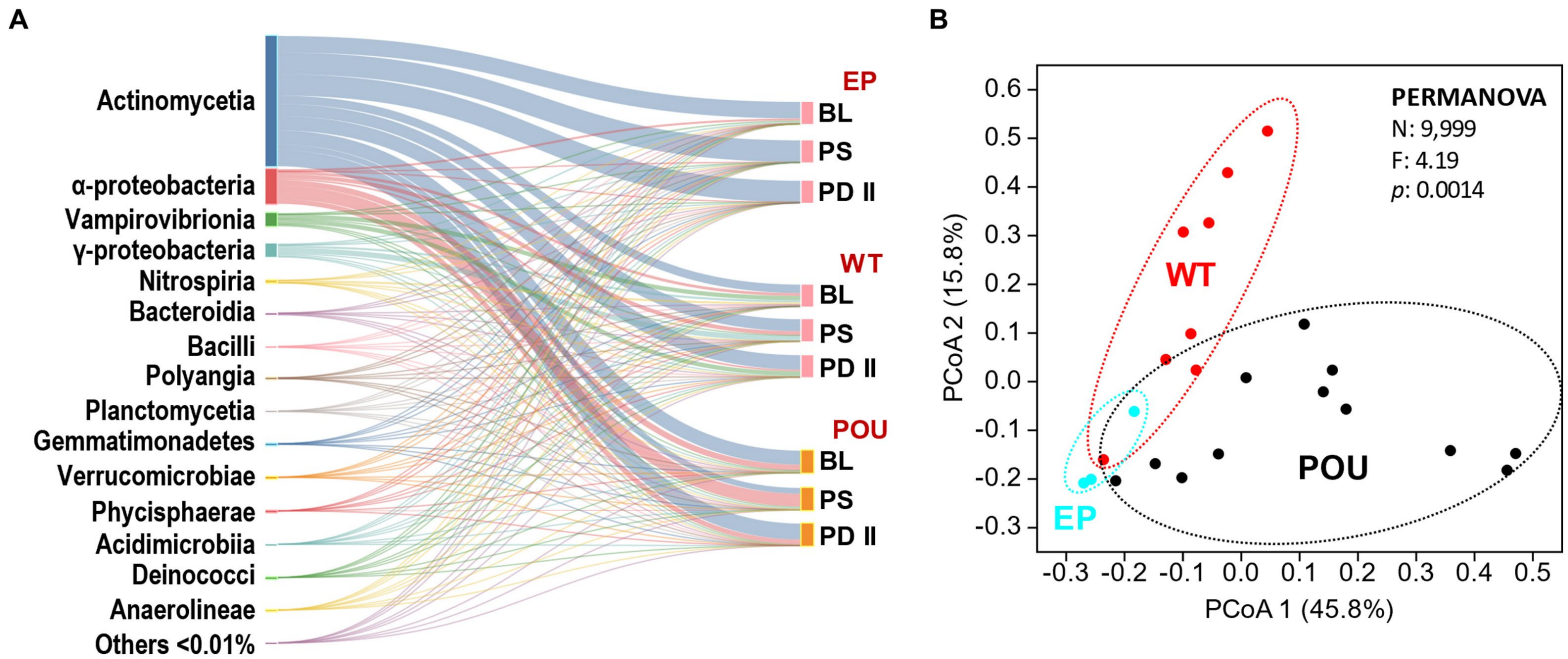
Comparison of EP, WT, and POU bacterial communities across flushing events. **(A)** Sankey diagram of the taxonomic distribution of bacteria at the class level. Classes are ordered from top to bottom by the most abundant in the total community. Others represent 214 additional classes with a relative distribution of <0.01%. The two columns from left to right represent the taxonomic groups scaled by the number of taxonomic representatives and flushing event for their respective sections. **(B)** PCoA of metagenomes based on Bray–Curtis similarity at the genus level. Sections: entry point (EP); water tank and recirculation (WT); point-of-use (POU). Flushing events: baseline (BL); post stagnation (PS); post drain II (PD II).

Twenty-three metagenome libraries from EP, WT, and POU were generated and analyzed for this study. Bulk water and biofilms were collected from 3 flushing events (BL, PS, and PD II). A total of 4,979 bacterial genera were identified using the Kraken2 package with the database GTDB release 207 (rarefied to 72,300,000 reads per HPS section). Only 1,336 bacterial genera (27% of the total genus diversity) were shared by EP, WT, and POU, representing 99.7–99.9% of the reads in their respective sections. The number of bacterial genera in the EP shared with the hot water sections is <161, while the number of genera shared between the WT and POU hot water sections increased to 1,362. Further analysis revealed that 134 (8% of the bacterial genera and 0.001% of reads), 868 (23 and 0.015%), and 989 (27 and 0.014%) genera were found exclusively in the EP, WT, and POU sections, respectively. Taxonomic classification revealed that most of the diversity was associated with the class *Actinomycetia* (68.875%), *Alphaproteobacteria* (17.600%), *Vampirovibrionia* (5.536%), *Gammaproteobacteria* (6.269%), *Nitrospiria* (0.114%), *Bacteroidia* (0.886%), *Bacilli* (0.105%), *Polyangia* (0.016%), *Planctomycetia* (0.078%), *Gemmatimonadetes* (0.400%), *Verrucomicrobiae* (0.009%), *Phycisphaerae* (0.015%), *Acidimicrobiia* (0.002%), *Deinococci* (0.018%), and *Anaerolineae* (0.003%) with additional representatives of 214 classes detected to a lesser extent (≤0.01% each) (Figure 3A).

Metagenomic-based alpha diversity indexes and rarefaction analysis revealed this system’s diversity of bacterial groups. Observed bacteria genera (Supplementary Figure S6A) and diversity metrics (Supplementary Figures S6B,C) were higher in hot water sections (WT and POU) compared to the EP communities. In hot water sections, observed indices in WT slightly decreased compared to POU but did not reach statistical significance (Kruskal–Wallis, Dunn’s test *p* > 0.05). Principal coordinated analysis (PCoA) analysis formed three defined clusters (PERMANOVA: *F* = 4.19, *p* = 0.0014; Figure 3B). The community composition (i.e., distribution) shifted markedly between HPS sections: the dominant class switched with an increase in the temperature from *Actinomycetia* (>95%) in EP communities to *Alphaproteobacteria*, *Gammaproteobacteria*, *Vampirovibrionia*, *Nitrospiria*, *Bacteroidia*, and *Gemmatimonadetes* in hot water sections. The POU communities (i.e., biofilms) were differentiated from the WT by an increase in the proportion of *Alphaproteobacteria* (from 11 to 41%) and *Bacteroidia* (0.4 to 2%), and a decrease in the proportion of *Actinomycetia* (62 to 48%), *Gammaproteobacteria* (15 to 4%), *Vampirovibrionia* (11 to 5%), and *Gemmatimonadetes* (1.1 to 0.1%). This dissimilarity is explained by a small number of genus-level taxa (29 out of 2,977 representing <1% of the genus) whose relative abundance varied significantly among the HPS sections (PERMANOVA: *F* = 4.19, *p* = 0.0014; rarefied to 249,000 reads per HPS sample). The 29 taxa are among the most abundant taxa (representing >94% of the total distribution of reads) and explains 90% of the dissimilarity within HPS sections. The *Mycobacterium* genus represents 62% of the total diversity. The dominant taxa (in the following distribution order) in EP communities were closely related to members of the genus *Mycobacterium* and QKMZ01 (class *Vampirovibrionia*). Representatives of the genus *Mycobacterium*, QKMZ01, UBA4763 (class *Alphaproteobacteria*), *Sphingomonas*, *Nitrospira*_F, and *Methyloversatilis* were overrepresented in the WT section. The POU section was dominated by the genus *Mycobacterium*, *Bradyrhizobium*, *Sphingomonas*, QKMZ01, *Reyranella*, *Afipia*, and *Sphingopyxis*.

### Cell density of OPPPs and FLA in drinking water samples

A total of 211 bulk water samples were analyzed for the presence of OPPPs along with two FLA (Supplementary Table S1). Across all assays, *L. pneumophila* was the most prevalent pathogen (86% of samples), followed by *M. intracellulare* (66%) and *P. aeruginosa* (21%). Interestingly, *M. avium* and *M. abscessus* were not detected in any samples. The data revealed a relatively low prevalence of *Acanthamoeba* spp. (4%), while *V. vermiformis* was widely detected (81%) across all the sampling locations within the HPS.

As summarized in Supplementary Table S2 and Supplementary Figure S7, *L. pneumophila,* and *V. vermiformis* had the highest level of cell density among the selected targets. The gene copy number (GCN) per mL was converted to log_10_(*x* + 1). The average of the estimated *L. pneumophila* gene copy number (±SD) is 1.4 ± 0.3, 2.7 ± 0.8, and 2.5 ± 1.0 copies for EP, WT, and POU, respectively. The *L. pneumophila* quantities in EP were significantly lower (Kruskal–Wallis, *H* = 38.1, Dunn’s test *p* < 0.0001) than those in WT and POU sections. Similarly, the *V. vermiformis* density in the EP section (0.8 ± 0.4 copies) was an order of magnitude lower than those in the hot water sections (ranged from 1.8 ± 0.9 to 2.5 ± 1.2 copies). Furthermore, significant differences in cell density were found between the hot water sections for *L. pneumophila* and *V. vermiformis*. In contrast, *M. intracellulare* quantities decreased significantly in hot water sections from 0.8 ± 0.3 to 0.4 ± 0.3 copies (Kruskal–Wallis, *H* = 18.6, Dunn’s test *p* < 0.001). For *M. intracellulare*, no significant differences in cell density were detected in both hot water sections. No significant difference was observed for *P. aeruginosa* copies (ranged from 0.2 ± 0.1 to 0.3 ± 0.2) among all sections (Kruskal-Wallis, *H* = 0.6, Dunn’s test *p* > 0.05).

**Figure 4:**
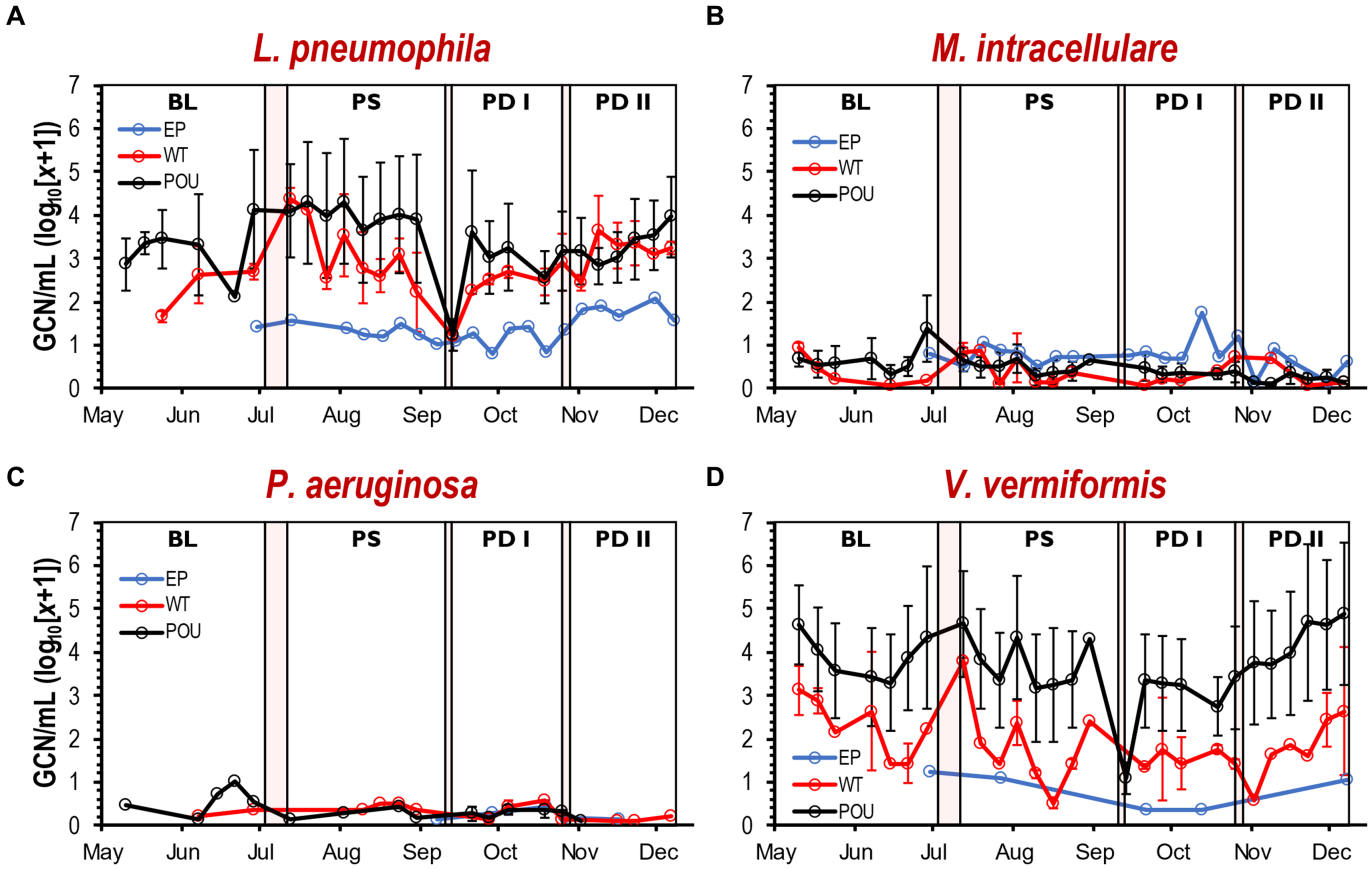
Cell density by qPCR of selected OPPPs and FLA in drinking water samples. Average gene copy number (log_10_[*x* + 1]/mL) detected for *L. pneumophila* **(A)**, *M. intracellulare* **(B)**, *P. aeruginosa* **(C)**, and *V. vermiformis* **(D)** at each section and flushing event. Clear and red boxes represent flushing events and simulated residential activities, respectively (see Figure 1). Sections: entry point (EP); water tank and recirculation (WT); point-of-use (POU). Flushing events: baseline (BL); post stagnation (PS); post drain I (PD I); post drain II (PD II). Bars represent ± SD. GCN, gene copy number.

Variation in GCN (i.e., cell density) was noticed between flushing events at each hot water section (Supplementary Figure S8). The Kruskal-Wallis one-way ANOVA test determined that changes in cell density for *M. intracellulare*, *P. aeruginosa*, and *V. vermiformis* were not different within flushing events (Supplementary Table S3). In contrast, cell density for *L. pneumophila* changed significantly between some of the flushing events in WT (Kruskal–Wallis, *H* = 8.6, Dunn’s test *p* < 0.0001) and POU (Kruskal–Wallis, *H* = 22.2, Dunn’s test *p* < 0.001) sections. Furthermore, no immediate effect was observed from the simulated residential flushing activity on cell density for most of the targeted species (Supplementary Figure S9). In most cases, the cell density remained consistent or recovered immediately to previous levels within a short time (Figure 4). As observed previously with the physico-chemical parameters, the cell density of selected species in both hot water sections followed similar patterns over time (Figure 4).

### Correlation between targeted species and environmental parameters

**Figure 5:**
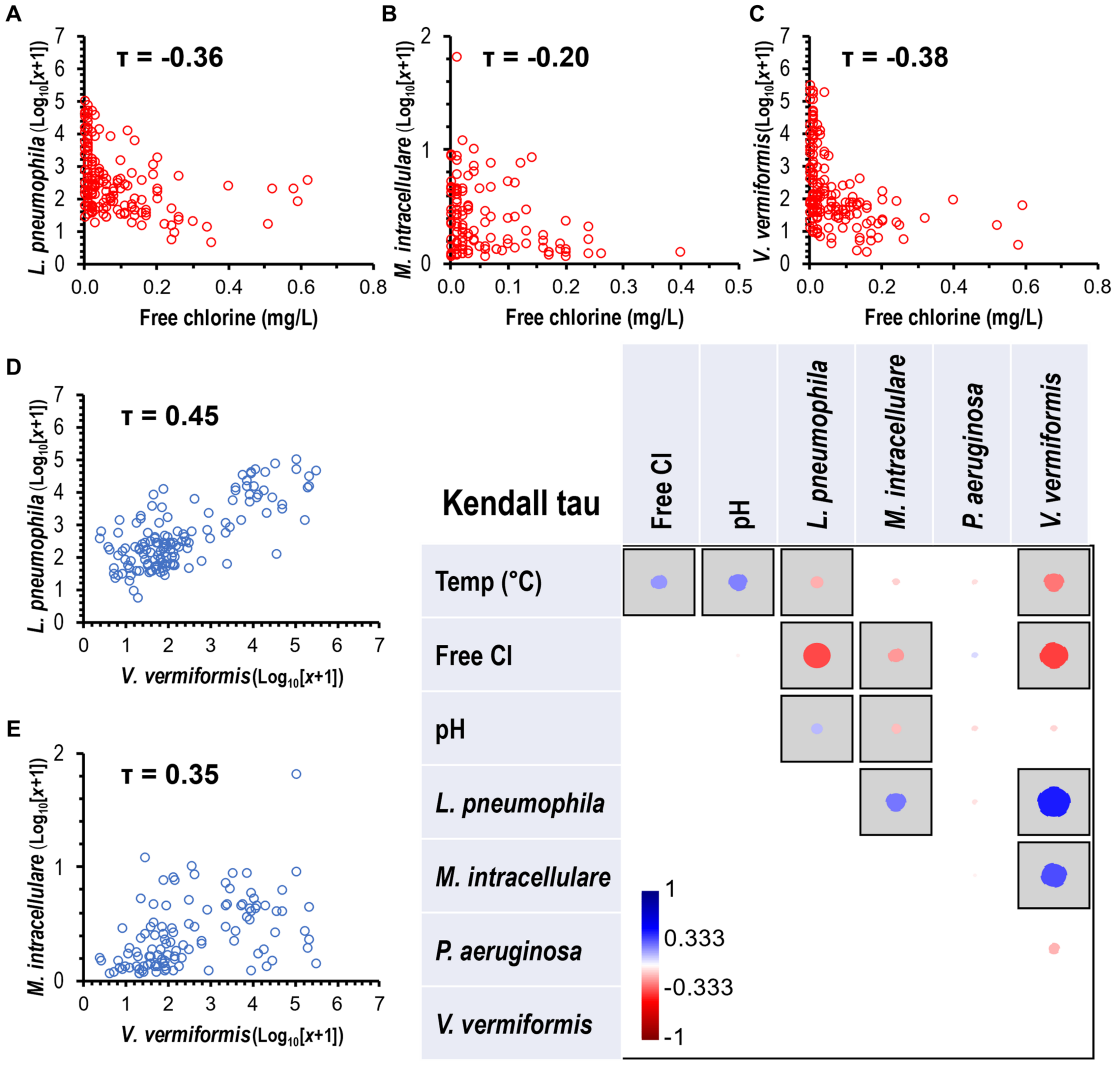
Correlation of OPPP taxa and water quality variables. Heatmap showing the Kendall’s Tau (τ) correlation coefficients and statistical significance between OPPP abundance and water physico-chemical parameters. Significant correlations marked by gray squares (*p* < 0.05) with positive and negative correlations displayed in blue and red circles, respectively. Size and color intensity is proportional to the correlation coefficients, as shown on the scale below the matrix. Kendall’s Tau negative correlations between disinfectant residual and **(A)** *L. pneumophila*, **(B)** *M. intracellulare*, and **(C)** *V. vermiformis*, and positive correlations between *V. vermiformis* and **(D)** *L. pneumophila* and **(E)** *M. intracellulare*.

The non-parametric Kendall’s tau (τ) correlation test showed an association between environmental parameters (temperature, chlorine, and pH) and targeted species (Figure 5). The species *L. pneumophila* (τ = −0.36, *p* < 0.0001, Figure 5A), *M. intracellulare* (τ = −0.20, *p* < 0.01, Figure 5B), and *V. vermiformis* (τ = −0.38, *p* < 0.0001, Figure 5C) were negatively correlated with an observed increase in chlorine residual. In addition, *L. pneumophila* (τ = −0.16, *p* < 0.01) and *V. vermiformis* (τ = −0.27, *p* < 0.0001) were negatively associated with an observed increase in temperature. The occurrence of *L. pneumophila* (τ = 0.45, *p* < 0.0001) and *M. intracellulare* (τ = 0.35, *p* < 0.0001) were significantly higher in samples where there was a substantial concentration of *V. vermiformis* (Figures 5D,E). The correlation analysis revealed a range of weak to moderate associations between environmental parameters and the occurrence of FLA in water samples.

## Discussion

The microbial community composition of the HPS simulator shows a highly diverse bacterial community with evidence of spatial and temporal structuring influenced by the environmental conditions (cold water entry point vs. hot water and bulk water vs. biofilm). Early studies using 16S-rRNA-gene-based high-throughput DNA sequencing of the same HPS indicated that the bacterial community structure of a hot water faucet was significantly different from those of a cold water faucet (Zhang et al., 2021). As noted above, the effect of water heater temperature is evident and have a substantial impact on the microbial communities (Meyer et al., 2023). This dissimilarity is explained by the 29 bacterial genera (out of 4,979) representing >94% of the total read distribution. The rest of the taxa represent the rare biosphere and can explain <10% of the dissimilarity within the HPS microbial community. This suggests minor contributions to the dissimilarities found within the community. Nevertheless, we must recognize the importance of this fraction and the possible implications for their contribution to the overall function of this diverse microbial ecosystem. Furthermore, additional investigations of the rare biosphere (including opportunistic pathogens that can cause infectious disease) suggest that these communities have the potential to seed other biofilm communities, which can increase under favorable conditions (Gomez-Alvarez et al., 2016).

The unique characteristics of premise plumbing can provide a favorable environment for the microbial proliferation of OPPPs and the establishment of biofilms within the premise plumbing (National Research Council, 2006). As a result, premise plumbing infrastructures can amplify the potential public health risk within the drinking water distribution system. The HPS is dominated by the class *Actinomycetia* with a high proportion consisting of the genus *Mycobacterium* and representatives of the species *M. phocaicum*, *M. gordonae*, *M. gadium*, *M. tusciae*, *M. paragordonae*, *M. mucogenicum*, and *M. chelonae*. The dominance of this class and their respective genus were not altered after changes in the flushing event (*circa* 2021). Community composition of samples obtained in 2012–2013 indicated the relative higher abundance of the family *Mycobacteriaceae* (approximately 30%) in cold and hot water samples (Zhang et al., 2021). It appears that environmental disturbances, as with any water management changes (i.e., flushing event), does not influence the prevalence and abundance of NTM in the HPS. This group of nontuberculous mycobacteria (NTM) are ubiquitous in the environment (Covert et al., 1999) and have been frequently found in residential premise plumbing (Dowdell et al., 2019). NTM has emerged as a significant cause of opportunistic infections causing pulmonary disease with their incidence, and the number of deaths associated with these infections have been steadily increasing globally (Ratnatunga et al., 2020). For specific populations of OPPPs, our analysis confirmed the widespread prevalence of *L. pneumophila* in 86% of bulk water samples, followed by *M. intracellulare* at 66% and *P. aeruginosa* at 21%. Interestingly, *M. avium* and *M. abscessus* were undetected in any samples. Similar results were obtained on samples collected in 2012–2013 by Lu et al. (2017a) with an overall occurrence of *Mycobacterium* spp. of 100%, followed by *Vermamoeba vermiformis* with 91%, *Legionella* spp. with 59%, *P. aeruginosa* with 14%, and *Acanthamoeba* spp. with 5%. These results clearly identify the HPS as a consistent reservoir for OPPPs (Hayward et al., 2022).

The microbial proliferation and growth within premise plumbing are directly associated with the complexity of the infrastructure, along with the various physico-chemical water quality parameters. For example, Ling et al. (2018) showed that small-diameter pipes widely used in residential homes harbored the highest cell density compared to the city-water supply microbiome. Water quality parameters such as chlorine concentration, pH (Ji et al., 2015), and water heater temperature (Ji et al., 2017) exhibited the strongest determinants of the microbial composition of the building plumbing microbiome. Early studies indicated significant variations in the density of OPPPs and water temperatures, especially for *Mycobacterium* spp., *Legionella* spp. and *V. vermiformis* (Lu et al., 2017a). This was observed in our HPS, where selected OPPPs and FLA were detected in higher abundances in the residential hot water sections than in entry point samples. The hot water sections were characterized by a lower chlorine residual, large surface area, and an increase in water temperature and pH. The only exception was *M. intracellulare,* with a greater increase in gene count observed at the entry point, but further qPCR analysis using mycobacterial marker genes (ITS and 23S rRNA) indicated a higher overall cell density of the genus *Mycobacterium* within all the residential sections (data not shown). On average, *L. pneumophila* and *V. vermiformis* showed an increase in one or more orders of magnitude. Among the hot water section, point-of-use (POU) devices showed a higher gene count for *L. pneumophila* and *V. vermiformis*. POU, such as the shower and faucets, are important locations for biofilm formation and bacterial regrowth (Hemdan et al., 2021; Hayward et al., 2022). Establishing within a multispecies biofilm is a strategy by *L. pneumophila* to overcome the nutrient limitation found in the environment (Declerck, 2010; Abu Khweek and Amer, 2018). In this case, adhering to a pre-established biofilm by other bacteria (e.g., NTM) assists in the survival and growth of *L. pneumophila* in the HPS. This is demonstrated by the results of the metagenome-based analysis indicating the presence and relatively high abundance of NTM in POU biofilm.

An interesting topic of discussion and research for ensuring public health in plumbing systems is the relationship between bacteria and FLA. Previous studies demonstrated that many bacteria, including OPPPs, benefit from interactions with FLA (Sun et al., 2018). The cyst stage of FLA is a survival mechanism that is resistant to various stresses and provides a protective niche for pathogenic bacteria against adverse conditions (Lambrecht et al., 2015). Indeed, our study indicated that the species *L. pneumophila* and *M. intracellulare* were positively correlated with an increase in the abundance of *V. vermiformis*. Previous studies have noted a positive association between the relative abundances of pathogenic bacteria and *V. vermiformis* (Siddiqui et al., 2021). *Mycobacterium* spp. and *V. vermiformis* not only co-occurred with *Legionella* spp. but also trended to increase with increasing temperatures (Lu et al., 2017a). Protozoa serves as habitats that provide the environmental host for the survival and replication of OPPP species in drinking water environmental settings (Malinowski et al., 2022; Nisar et al., 2022). Furthermore, our study suggested that these interactions are determined by the location or type of environment in the HPS. Our observation shows that the relationship between *L. pneumophila* and *V. vermiformis* increased significantly in POU devices (τ = 0.60) compared to the WT section (τ = 0.45). Comparable results were observed for *Mycobacterium* spp. (WT τ = 0.32 vs. POU τ = 0.42). POU devices such as the shower and faucets are important locations for biofilm formation and bacterial regrowth. These locations are characterized by small-diameter pipes, and high surface area that is considered the preferred niche for the interactions of bacteria and potential amoeba hosts. Interestingly, *M. intracellulare* maintains a similar association in both sections. This may suggest that the association between *M. intracellulare* and FLA is not influenced by the location in the HPS. Conversely, the *L. pneumophila* gene copy number was positively associated with those of *M. intracellulare*. Similar observations from a drinking water distribution system have been reported in the literature (Lu et al., 2016). Therefore, their association, particularly in HPS, may be more important than previously considered. Overall, the relationship between OPPPs and FLA and their locations in the plumbing system is of increasing concern for facility managers and residential occupants as they cause human diseases. Favorable conditions (e.g., residual disinfectant, water temperature, stagnation) found at these POU locations promoted the establishment and further interaction of OPPPs and FLA. Consequently, drinking water taps (i.e., POU) may be an important source of human exposure to aerosols containing OPPPs in residential and building structures (Hayward et al., 2022).

The growth of bacterial pathogens in premise plumbing may be unavoidable; however, understanding the effect of residential activities and the potential response by OPPP populations is important for ensuring public health. There is a common notion that episodes of disturbances (e.g., residential activities) induce a selection pressure on microbial populations (Allison and Martiny, 2008); however, our data provides evidence that residential activities do not produce long-term effects on cell density and microbial community structure. This study demonstrated that targeted OPPP and FLA populations, although sensitive to changes due to operational parameters (i.e., stagnation, drain), responded to a disturbance by returning to their stable state after a brief shift in cell abundance (i.e., resilience). After stagnation (at least 2 weeks), OPPP and amoeba densities immediately increased and gradually decreased to the baseline. This suggests maintaining disinfectant residuals is essential for ensuring low microbial numbers and diversity. Furthermore, monitoring the microbial population after drainage of the hot water tank and partial drainage of the entire HPS demonstrated no significant mitigation of the selected OPPPs. This study demonstrates that these organisms can adjust to their environment during such events and may survive in biofilms and/or grow within FLA, protecting them from stressors in the supplied water.

## Conclusion

The microbial communities in the HPS sections exhibited a distribution pattern of few dominant taxa and a significant representation of low-abundance species.

Metagenomes-based analysis confirmed that premise plumbing (i.e., hot water sites) harbors higher microbial richness and composition compared to communities analyzed from the point-of-entry (i.e., water main). The observed changes in the community structure corresponded to reduction of chlorine residual and changes in water temperature, suggesting that these conditions should induce changes on the microbial community.

Community composition associated with deterioration of physico-chemical water quality was not only evident at the community structure level, but also evident at the population level (e.g., OPPPs).

The effect of residential activity combined with the resilience of a diverse group of opportunistic pathogens may constitute a significant challenge for the efficiency of drinking water management plans and affect drinking water safety.

Comparative analysis of the community suggested the relevance of co-occurrence relationships in the drinking water microbial composition of HPS. The presence and occurrence of FLA may have significant implications for the survival of OPPPs in residential systems.

## Data availability statement

The datasets presented in this study can be found in online repositories. The names of the repository/repositories and accession number(s) can be found in the NCBI Sequence Read Archive (SRA) under the BioProject PRJNA961987 with the following BioSample numbers: SAMN34376234, SAMN34376235, SAMN34376236, SAMN34376237, SAMN34376238, SAMN34376239, SAMN34376240, SAMN34376241, SAMN34376242, SAMN34376243, SAMN34376244, SAMN34376245, SAMN34376246, SAMN34376247, SAMN34376248, SAMN34376249, SAMN34376250, SAMN34376251, SAMN34376252, SAMN34376253, SAMN34376254, SAMN34376255, and SAMN34376256.

## Author contributions

VG-A: Investigation, Formal Analysis, Visualization, Writing – original draft, Writing – review & editing. HR: Conceptualization, Investigation, Formal Analysis, Data curation, Writing – original draft, Writing – review & editing. MT: Investigation, Writing – review & editing. MM: Investigation, Writing – review & editing. CM: Investigation, Writing – review & editing. MU: Investigation, Writing – review & editing. DW: Investigation, Writing – review & editing. DL: Conceptualization, Investigation, Writing – review & editing. LB: Conceptualization, Investigation, Formal Analysis, Data curation, Writing – original draft, Writing – review & editing.

## Footnotes

^1^http://sourceforge.net/projects/bbmap

^2^http://ftp.tue.mpg.de/ebio/projects/struo2/GTDB_release207/kraken2

^3^https://sankeymatic.com/

## Abbreviations

BL: Baseline (flush protocol)
EP: Entry point
F: Faucet
FLA: Free-living amoeba
HPS: Home plumbing system
LCR: Lead and copper rule
NTM: Nontuberculous mycobacterial
OPPP: Opportunistic premise plumbing pathogens
PD: Post drain (flush protocol)
POU: Point of use
PS: Post stagnation (flush protocol)
SH: Shower head
WT: Water tank
