# Supplementary material for: Assessing residential activity in a home plumbing system simulator: monitoring the occurrence and relationship of major opportunistic pathogens and phagocytic amoebas
Source: Front Microbiol. 2023 Oct 17;14:1260460. doi: 10.3389/fmicb.2023.1260460 (PMC10616306; doi:10.3389/fmicb.2023.1260460)
Supplement: Supplementary file 1 [file Data_Sheet_1.pdf]

Supplementary Material

Assessing residential activity in a home plumbing system simulator: monitoring the occurrence and relationship of major opportunistic pathogens and phagocytic amoebas

Vicente Gomez-Alvarez[1*], Hodon Ryu[1], Min Tang[2, #], Morgan McNeely[1], Christy Muhlen[1], Megan Urbanic[2], Daniel Williams[1], Darren Lytle[1], and Laura Boczek[1]

[1]U.S. Environmental Protection Agency, Office of Research and Development, Cincinnati, Ohio 45268

[2]Oak Ridge for Science and Education Research Fellow at U.S. Environmental Protection Agency, Cincinnati, Ohio 45268

[#]Current affiliation: CDM Smith, 11500 Northlake Dr. Cincinnati, Ohio 45249

Table of Contents

		Page
**Supplementary Materials and Methods**		S2
**Tables**		
Table S1	Primers and probes for qPCR assays	S4
Table S2	Water quality and gene copy number in HPS sections	S5
Table S3	Water quality and gene copy number in hot water	S6
**Figures**		
Figure S1	Diagram of home plumbing system (HPS) simulator	S7
Figure S2	Physico-chemical parameters in HPS sections	S8
Figure S3	Water temperature in hot water sections and flushing events	S9
Figure S4	Free chlorine residual in hot water sections and flushing events	S10
Figure S5	pH in hot water sections and flushing events	S11
Figure S6	Bacterial richness and community diversity	S12
Figure S7	Cell density by qPCR analysis of targeted species in HPS sections	S13
Figure S8	Cell density by qPCR analysis of targeted species at each flushing event	S14
Figure S9	Effect of residential activity on selected OPPPs and FLA	S15
**References**		S16

MATERIALS and METHODS

Home Plumbing System (HPS) Simulator

The HPS simulator was constructed on January 23, 2012 (Lytle et al., 2021) and contained approximately 56 m Type M copper pipes (inside diameter 1.45 cm), a flow meter totalizer (located at the start of the simulator and recorded the flow rate of the whole simulator), a electric water heater, dishwasher, washing machine, a shower head, and four faucets. The water tank, shower head, toilet, and four faucets are connected via copper piping, brass fixtures, and solder joints. Faucet 1 is a cast brass utility faucet. Faucets 2 to 4 are three identical bathroom-type hot-and-cold water faucets with a chrome exterior and a cast brass and plastic interior. The HPS systems was designed with a hot and cold water pipe system. The lengths of the cold water line (in meters) from the flow meter to Faucet 1, Faucet 2, Faucet 3, Faucet 4, and the shower were 16.6, 14.8, 12.0, 9.1, and 15.5, respectively. The lengths of the hot water line (in meters) from the water heater to Faucet 1, Faucet 2, Faucet 3, Faucet 4, and the shower were 18.4, 16.1, 13.2, 10.2, and 16.4, respectively. Additional HPS simulator components included brass ball valves, brass check valves, bathtub, and a toilet.

The HPS was operated under a new "Random Reduced Normal Use" flushing protocol to simulate the average daily water use for a typical household of four residents (target total daily water use 70-80 gallons [≈265-303 L]). The faucets were given specific usage-type designations: three-bathroom taps (faucet 1, 2, and 4) and a kitchen tap (faucet 3). A dishwasher and washing machine where added to the HPS system. Household activities such as filling a glass of water, flushing the toilet, showering or washing dishes were mimicked. The cold water lines and the hot water tank are fed with water that is supplied by the building water supply (Greater Cincinnati Water Works tap water); a water chemistry of pH 7.7 ± 0.3, alkalinity 76 ± 14 mg $CaCO_3$/L, dissolved inorganic carbon 19 ± 4 mg C/L, calcium 28 ± 9 mg Ca/L, magnesium 8.4 ± 3.1 mg Mg/L, nitrate 0.88 ± 0.22 mg N/L, total phosphate 0.72 ± 0.57 mg PO_4/L, and sulfate 58 ± 22 mg SO_4/L during the study duration. The water heater generated hot water with an initial temperature set at 120°F (≈49°C). The target water use was achieved by manually flushing the hot and cold water for all faucets including the shower several times per weekday in a random schedule to

simulate a typical home usage. Dishwasher and washing machine are run once a week. The toilet is flushed on a random schedule daily.

Quantitative polymerase chain reaction (qPCR) analyses

Culture-independent qPCR analyses were conducted for three major OPPP groups: *Legionella pneumophila*, nontuberculous mycobacterial species (e.g., *Mycobacterium avium*, *M. intracellulare*, and *M. abscessus*), and *Pseudomonas aeruginosa* and two amoebas (*Acanthamoeba* and *Vermamoeba vermiformis*) (Table S1). A SYBR green qPCR assay was performed for *V. vermiformis* as described by Ryu et al., 2013. The assay was performed in a 25-μL reaction mixtures containing 1× Power SYBR green master mix (Applied Biosystems, Foster City, CA), 0.4 μM of each primer (final concentration), and 2 μL of the template. The amplification protocol involved an initial incubation at 50°C for 2 min, followed by 95°C for 10 min and 40 cycles of 95°C for 15 s and an annealing temperature of 56°C for 1 min (Table S1). Taqman qPCR assays were performed in a 25-μL reaction mixture containing 1× TaqMan universal PCR master mix (Applied Biosystems, Foster City, CA, USA), 0.4 μM of each primer (final concentration) and 0.2 μM of a 6-FAM (6-carboxyfluorescein)-labeled hydrolysis probe (final concentration), and 2 μL of the template. The amplification protocol involved 40 cycles of 95°C for 15 s and optimum annealing temperature for 1 min (Table S1).

Reaction mixtures were prepared in MicroAmp Optical 96-well reaction plates with MicroAmp Optical Caps (Applied Biosystems, Foster City, CA). All qPCR assays were performed using a QuantStudio™ 6 Flex system (Applied Biosystems, Foster City, CA), followed by a melting curve analysis (i.e., from the annealing temperature to 90°C in 0.1-degree increments). Disassociation curves from the melting curve analysis were examined to determine the presence of potential primer-dimers and other non-specific reaction products. Signal intensity values were recorded for those reactions showing one corresponding amplification peak within the disassociation curves.

Two independent standard curves for each qPCR assay were generated by plotting threshold cycle (CT) values against the number of target gene copies corresponding to six 10-fold dilutions in duplicate of gBlock standards (IDT, Coralville, IA, USA). The target gene copy numbers (T) were estimated by the following equation:

$$T = [D/(PL \times 660)] \times 6.022 \times 10^{23}$$

where D (g/µL) is gBlock concentration, and PL (bp) is gBlock length in base pairs (Ryu et al., 2013). Percent amplification efficiencies were calculated by the instrument manufacturer's instructions (Applied Biosystems, Foster City, CA). Two no-template controls per PCR plate were used to check for cross-contamination.

Data availability

The raw sequence reads have been submitted to the NCBI Sequence Read Archive (SRA) under the BioProject PRJNA961987 with the following BioSample numbers: SAMN34376234, SAMN34376235, SAMN34376236, SAMN34376237, SAMN34376238, SAMN34376239, SAMN34376240, SAMN34376241, SAMN34376242, SAMN34376243, SAMN34376244, SAMN34376245, SAMN34376246, SAMN34376247, SAMN34376248, SAMN34376249, SAMN34376250, SAMN34376251, SAMN34376252, SAMN34376253, SAMN34376254, SAMN34376255, and SAMN34376256.

TABLES

Table S1 The sequences of primers and probes of qPCR assays.

**Target species**	**Primer sequence** (5'-3')	**Amplicon size** (bp)	**Annealing temp** (°C)	**References**	**Target genes**
L. pneumophila	Lpneu F1: CGGAATTACTGGGCGTAAAGG<br>Lpneu R1: GAGTCAACCAGTATTATCTGACCG<br>Lpneu P1: FAM-AAGCCCAGGAATTTCACAGAT−TAMRA	100	60	Donohue et al, 2014, 2019	16S rRNA
M. avium	MA/MI-F: GGGTGAGTAACACGTGTGCAA<br>MA-R: CCAGAAGACATGCGTCGTGA<br>MA/MI-P: FAM-TGCACTTCGGGATAAGCCTGGGAAA-TAMRA	97	60	Chern et al., 2015	16S rRNA
M. intracellulare	MA/MI-F: GGGTGAGTAACACGTGTGCAA<br>MI-R: CCACCTAAAGACATGCGACTAAA<br>MA/MI-P: FAM-TGCACTTCGGGATAAGCCTGGGAAA-TAMRA	100	60	Chern et al., 2015	16S rRNA
M. abscessus	MAb-F: CGATAGAGGACTTCGCCTAACC<br>MAb-R: TCGAGCACGTAAACTCCCTTTC<br>MAb-P: FAM-CCACTGACCGAACATCTATCCCGC-TAMRA	77	60	Steindor et al., 2015	*rpo*B
P. aeruginosa	ecfx-F: CGCATGCCTATCAGGCGTT<br>ecfx-R: GAACTGCCCAGGTGCTTGC<br>ecfx-TM: FAM- ATGGCGAGTTGCTGCGCTTCCT-TAMRA	64	60	Anuj et al., 2009	*ecfx*
Acanthamoeba spp.	TaqAcF1: CGACCAGCGATTAGGAGACG<br>TaqAcR1: CCGACGCCAAGGACGAC<br>TaqAcP1: FAM-TGAATACAAAACACCACCATCGGCGC-TAMRA	65	60	Riviere et al., 2006	18S rRNA
V. vermiformis	Hv1227F: TTACGAGGTCAGGACACTGT<br>Hv1728R: GACCATCCGGAGTTCTCG	502	56	Kuiper et al., 2006; Lu et al., 2015	18S rRNA

FAM, 6-carboxyfluorescein, fluorescence reporter dye; TAMRA, 6-carboxytetramethylrhodamine, fluorescence quencher dye

Table S2 Average physico-chemical water quality and gene copy number (GCN) of targeted species at each home plumbing system (HPS) section. BDL: below detection limit.

**Parameter**	**Samples** (n)	**HPS section**[†] (avg ±SD)		
		**EP**	**WT**	**POU**
**Physico-chemical**				
Temperature (°C)	210	22.1 ± 0.9	31.3 ± 4.3	25.5 ± 4.9
Free Chlorine (mg Cl_2/L)	210	0.86 ± 0.16	0.11 ± 0.14	0.07 ± 0.10
pH	208	7.6 ± 0.4	8.6 ± 0.5	8.2 ± 0.5
**Target species** (GCN [$\log_{10} x$+1])				
L. pneumophila	180	1.4 ± 0.3	2.7 ± 0.8	2.5 ± 1.0
M. avium	--	BDL	BDL	BDL
M. intracellulare	138	0.8 ± 0.3	0.4 ± 0.3	0.4 ± 0.3
M. abscessus	--	BDL	BDL	BDL
P. aeruginosa	45	0.2 ± 0.1	0.3 ± 0.2	0.3 ± 0.2
Acanthamoeba spp.	9	0.17 ± 0.0	0.3 ± 0.0	0.1 ± 0.1
V. vermiformis	170	0.8 ± 0.4	1.8 ± 0.9	2.5 ± 1.2

[†]HPS section: EP (entry point); WT (water tank and recirculation); POU (point-of-use).
Avg: average; SD: standard deviation.

Table S3 Average physico-chemical water quality and gene copy number (GCN) of selected opportunistic premise plumbing pathogens (OPPPs) and free-living amoeba (FLA) species at each flushing event in hot water samples.

Parameter	**Flushing event** (avg ±SD)			
	**Baseline**	**Post stagnation**	**Post drain I**	**Post drain II**
**Water Tank and Recirculation (WT)**				
**Physico-chemical**				
Temperature (°C)	32.0 ± 3.3	30.7 ± 2.9	31.3 ± 2.9	31.3 ± 7.5
Free Chlorine (mg Cl_2/L)	0.03 ± 0.02	0.15 ± 0.13	0.14 ± 0.09	0.12 ± 0.23
pH	8.4 ± 0.4	8.4 ± 0.4	8.5 ± 0.2	9.2 ± 0.2
**Target species** (GCN [$\log_{10} x$+1])				
L. pneumophila	2.2 ± 0.6	2.7 ± 1.0	2.5 ± 0.3	3.1 ± 0.5
M. intracellulare	0.5 ± 0.4	0.4 ± 0.4	0.3 ± 0.3	0.3 ± 0.3
P. aeruginosa	0.3 ± 0.1	0.4 ± 0.1	0.3 ± 0.2	0.1 ± 0.1
V. vermiformis	2.2 ± 0.8	1.9 ± 1.1	1.4 ± 0.5	1.7 ± 0.8
**Point-of-Use (POU)**				
**Physico-chemical**				
Temperature (°C)	25.6 ± 4.7	25.7 ± 4.5	24.5 ± 4.3	25.9 ± 6.3
Free Chlorine (mg Cl_2/L)	0.02 ± 0.01	0.10 ± 0.09	0.08 ± 0.07	0.07 ± 0.15
pH	8.0 ± 0.5	8.1 ± 0.4	8.1 ± 0.2	8.7 ± 0.4
**Target species** (GCN [$\log_{10} x$+1])				
L. pneumophila	2.7 ± 0.9	2.4 ± 1.3	2.2 ± 0.9	2.7 ± 0.7
M. intracellulare	0.5 ± 0.4	0.4 ± 0.3	0.3 ± 0.2	0.2 ± 0.1
P. aeruginosa	0.6 ± 0.3	0.3 ± 0.1	0.3 ± 0.1	0.2 ± 0.1
V. vermiformis	2.7 ± 1.2	2.3 ± 1.3	2.2 ± 0.9	2.5 ± 1.4

Avg: average; SD: standard deviation.

FIGURES

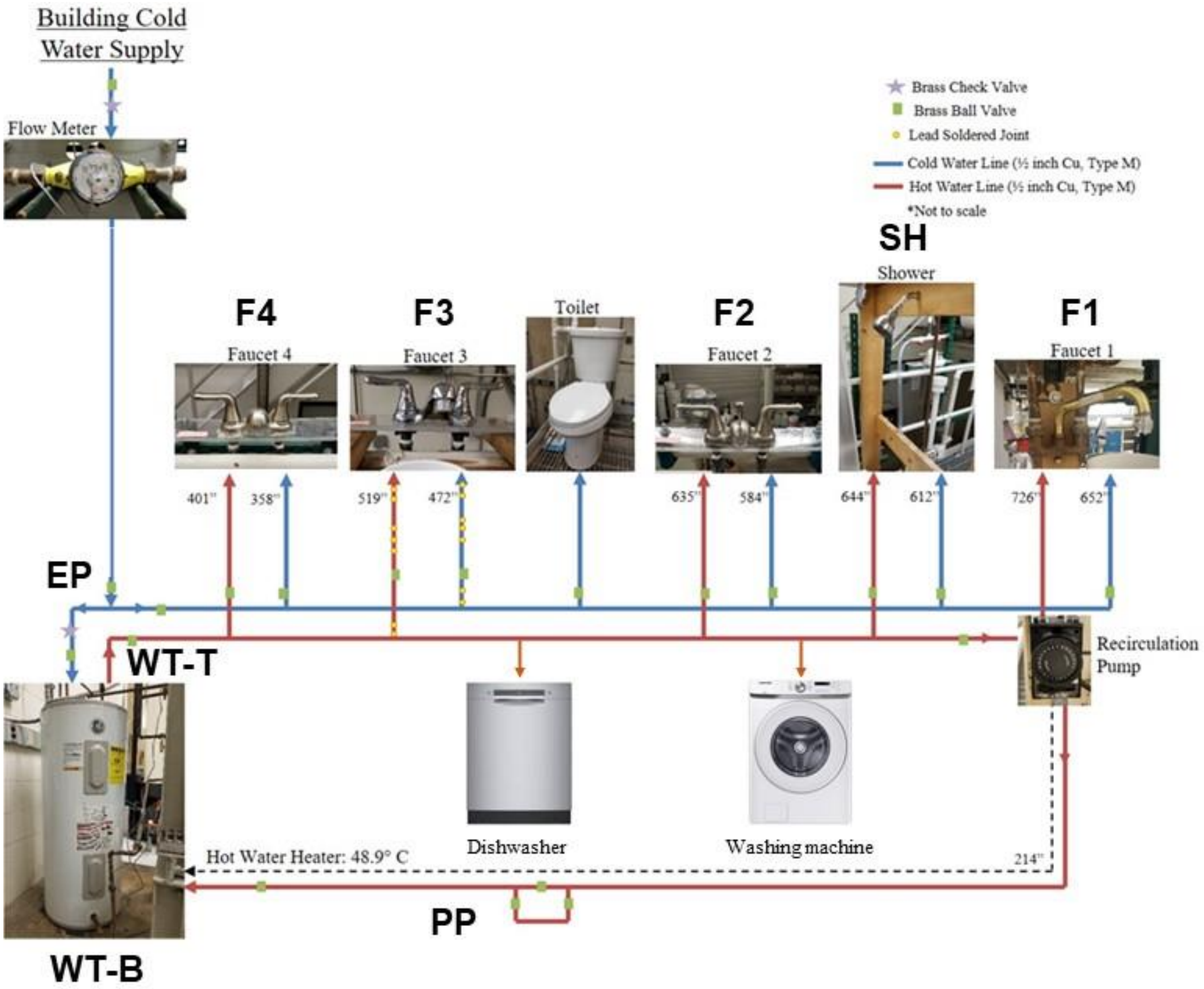

Figure S1 Home plumbing system (HPS) simulator. Schematic of the HPS and sample port locations for hot (—) and cold water (—). Sample ports: cold-water entry point (EP), hot water tank point of entry and return (WT-T and WT-B, respectively), premise plumbing recirculation (PP), faucets (F1, F2, F3, and F4), and the shower (SH).

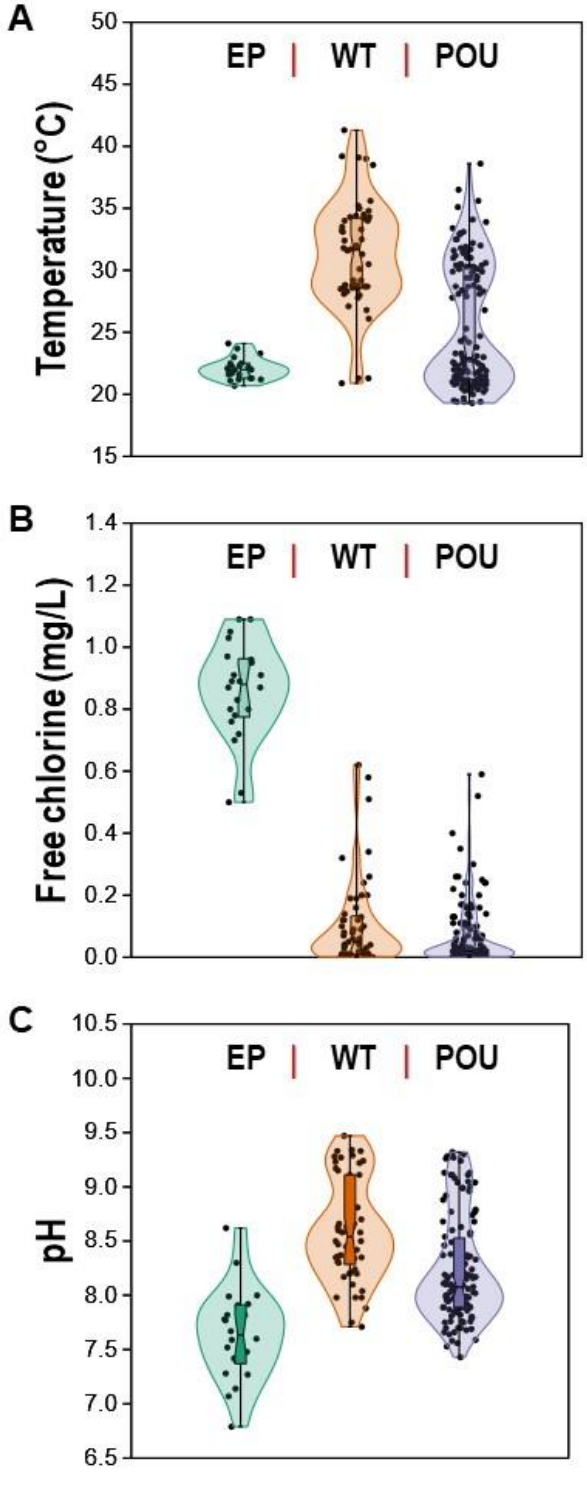

Figure S2 Water quality parameters in home plumbing system (HPS) sections. Violin plots showing measurements and concentrations of (A) temperature, (B) free chlorine residual, and (C) pH at each section and flushing event. Each dot represents a single sample event. Sections: entry point (EP); water tank and recirculation (WT); point-of-use (POU).

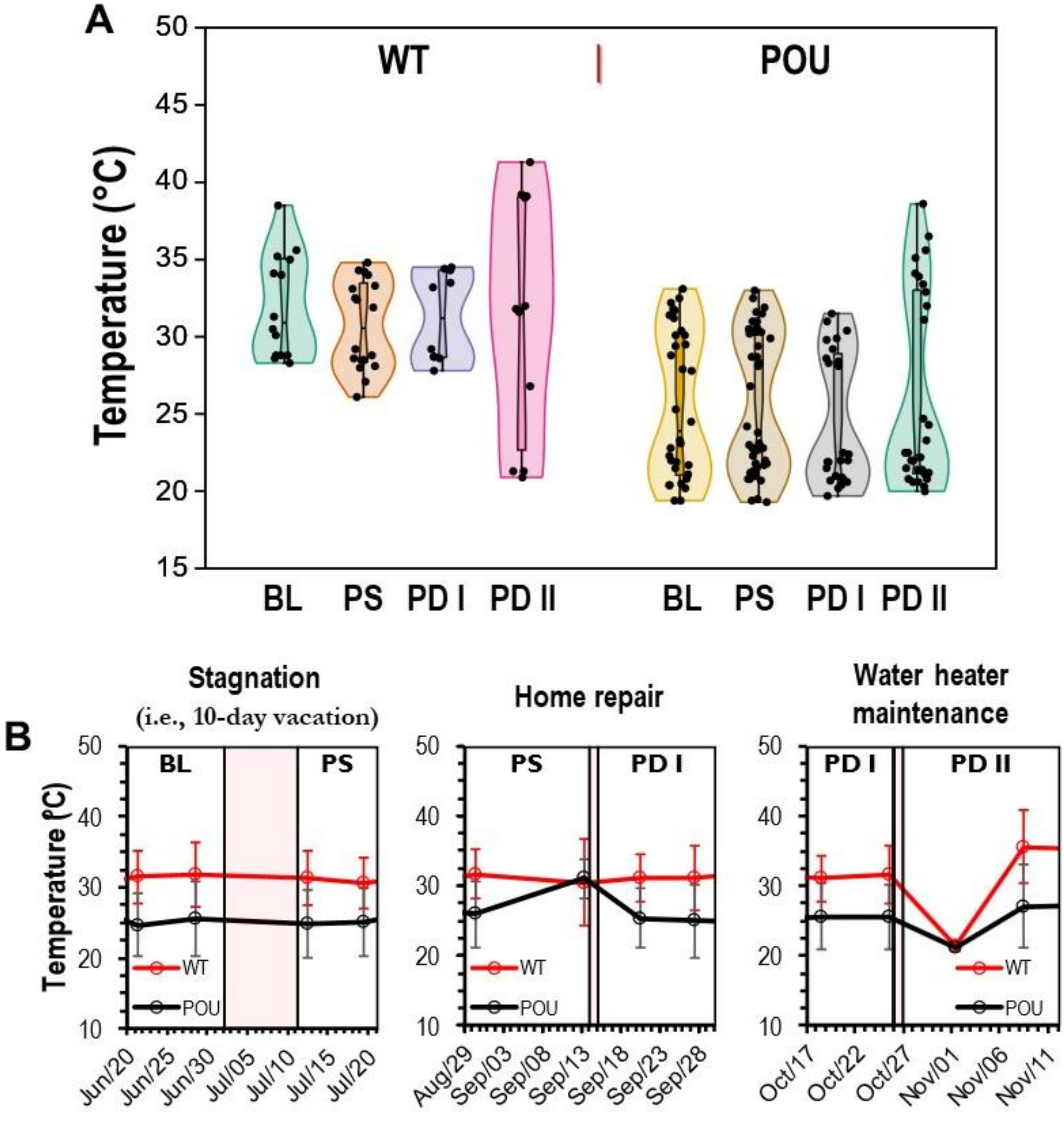

Figure S3 Measured water temperature in hot water sections. (A) Violin plots showing temperature at each flushing event in respective section. Sections: water tank and recirculation (WT); point-of-use (POU). Each dot represents a single sample event. (B) Effect of simulated residential activities (red boxes, see Figure 1) on water temperature. Flushing events: baseline (BL); post stagnation (PS); post drain I (PD I); post drain II (PD II). Bars represent standard deviation (±SD).

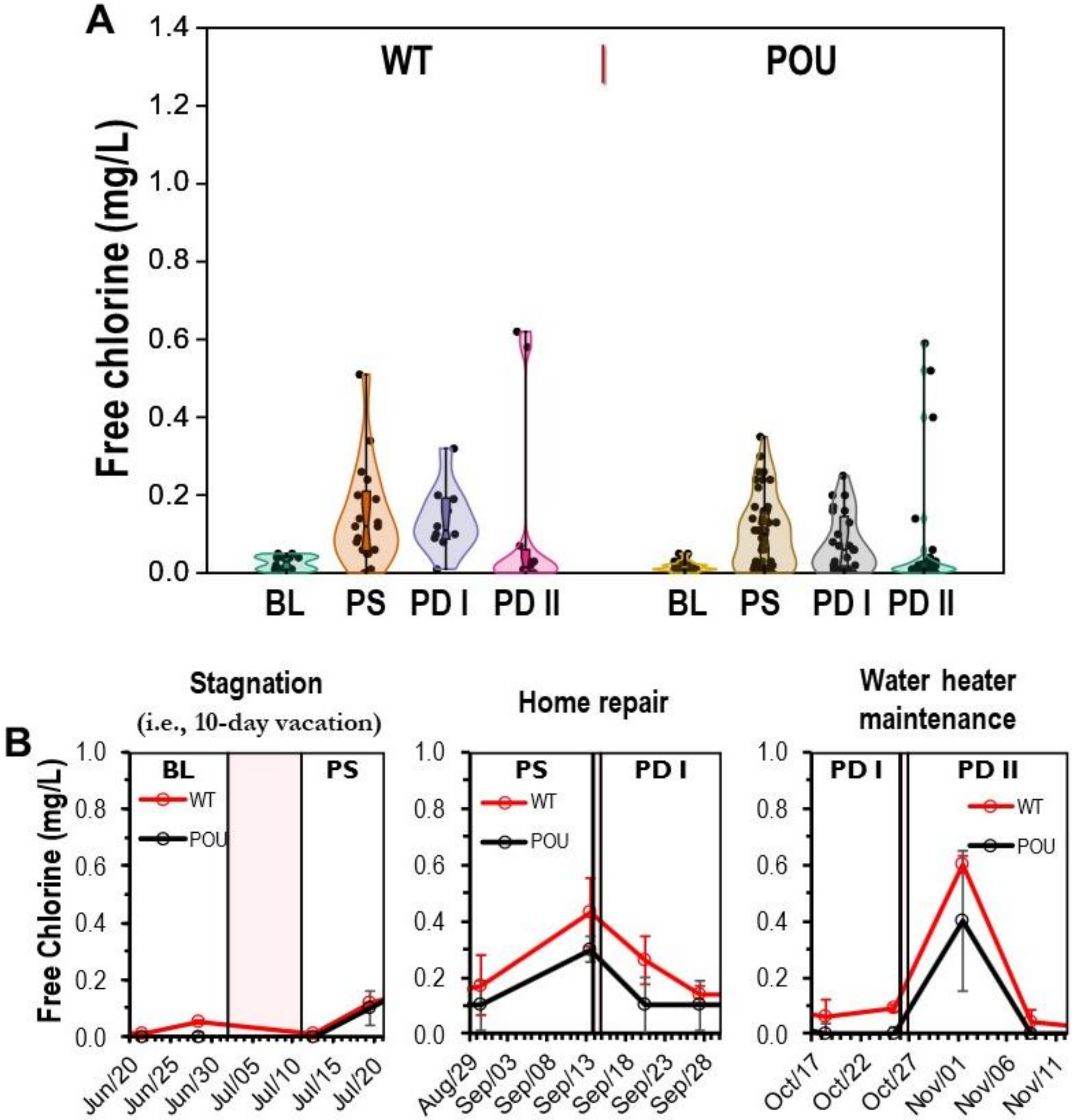

Figure S4 Measured free chlorine residual in hot water sections. (A) Violin plots showing chlorine residual at each flushing event in respective section. Sections: water tank and recirculation (WT); point-of-use (POU). Each dot represents a single sample event. (B) Effect of simulated residential activities (red boxes, see Figure 1) on chlorine residual. Flushing events: baseline (BL); post stagnation (PS); post drain I (PD I); post drain II (PD II). Bars represent standard deviation (±SD).

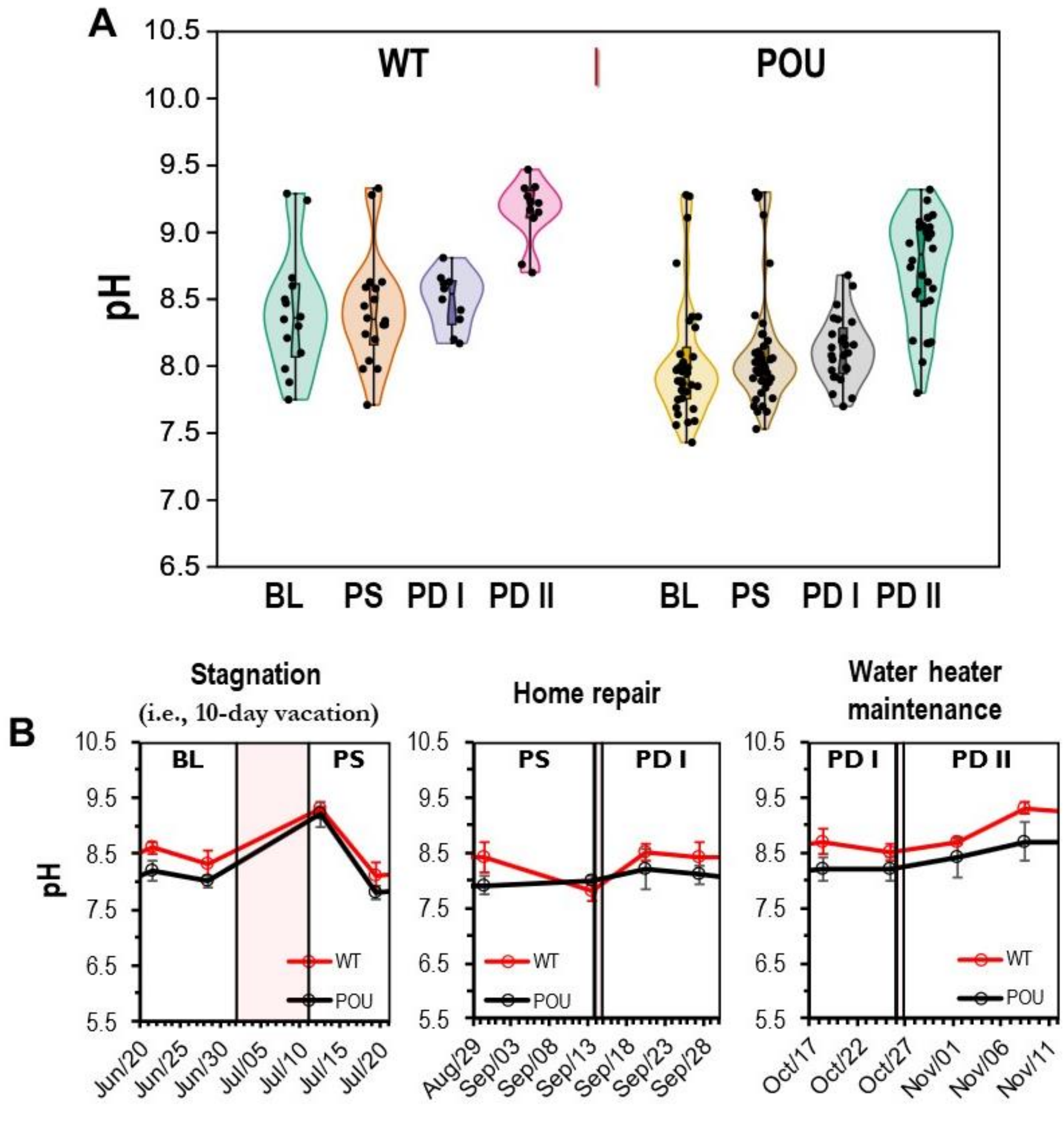

Figure S5 Measured pH in hot water sections. (A) Violin plots showing chlorine residual at each flushing event in respective section. Sections: water tank and recirculation (WT); point-of-use (POU). Each dot represents a single sample event. (B) Effect of simulated residential activities (red boxes, see Figure 1) on pH. Flushing events: baseline (BL); post stagnation (PS); post drain I (PD I); post drain II (PD II). Bars represent standard deviation (±SD).

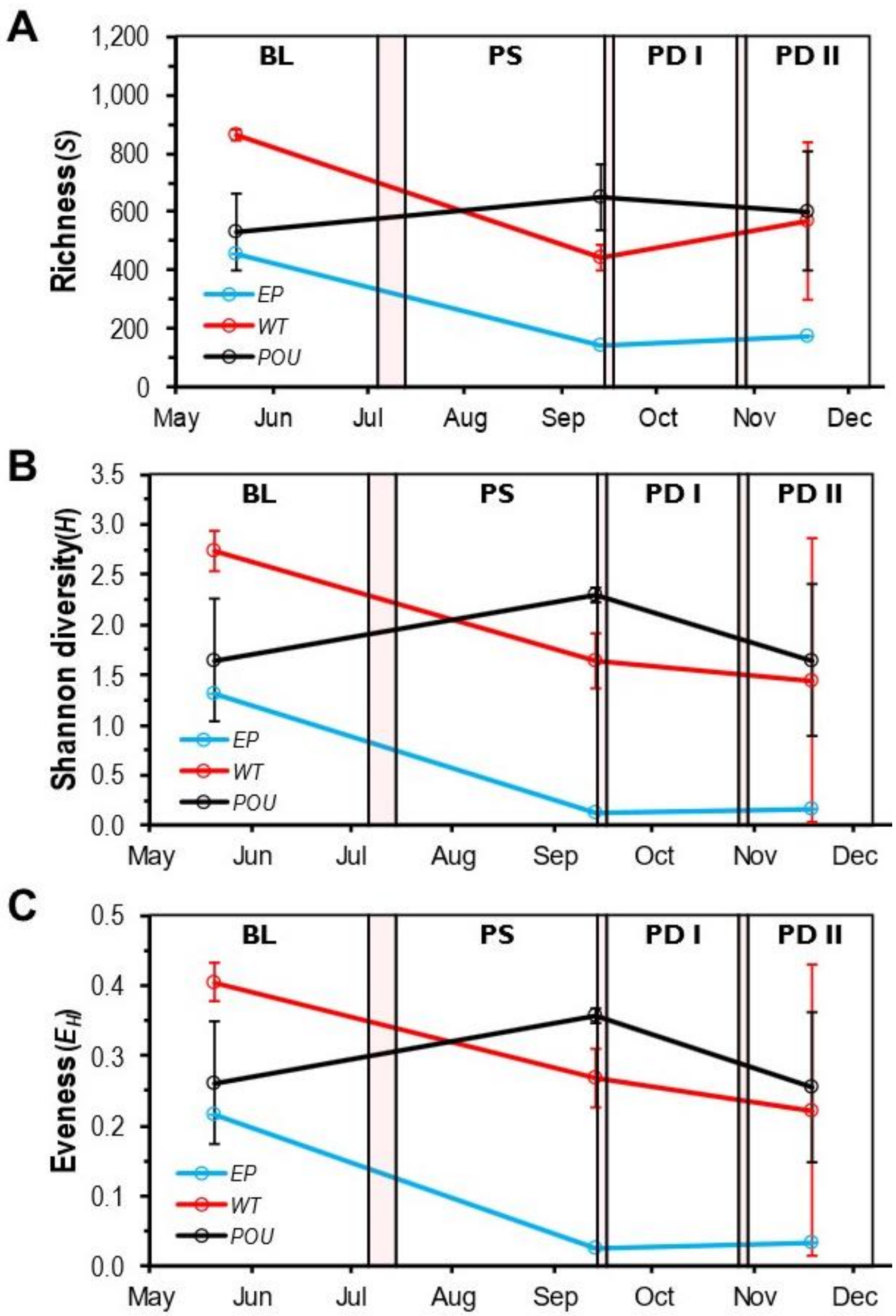

Figure S6 Bacterial richness and community diversity. Graph showing (A) richness (*S*), (B) Shannon-Wiener diversity (*H*), and (C) evenness (E_H) indices for sections in the HPS. Red and clear areas represent simulated residential activities and flushing events, respectively (see Figure 1). Sections: entry point (EP); water tank and recirculation (WT); point-of-use (POU). Flushing events: baseline (BL); post stagnation (PS); post drain I (PD I); post drain II (PD II). Bars represent standard deviation (±SD).

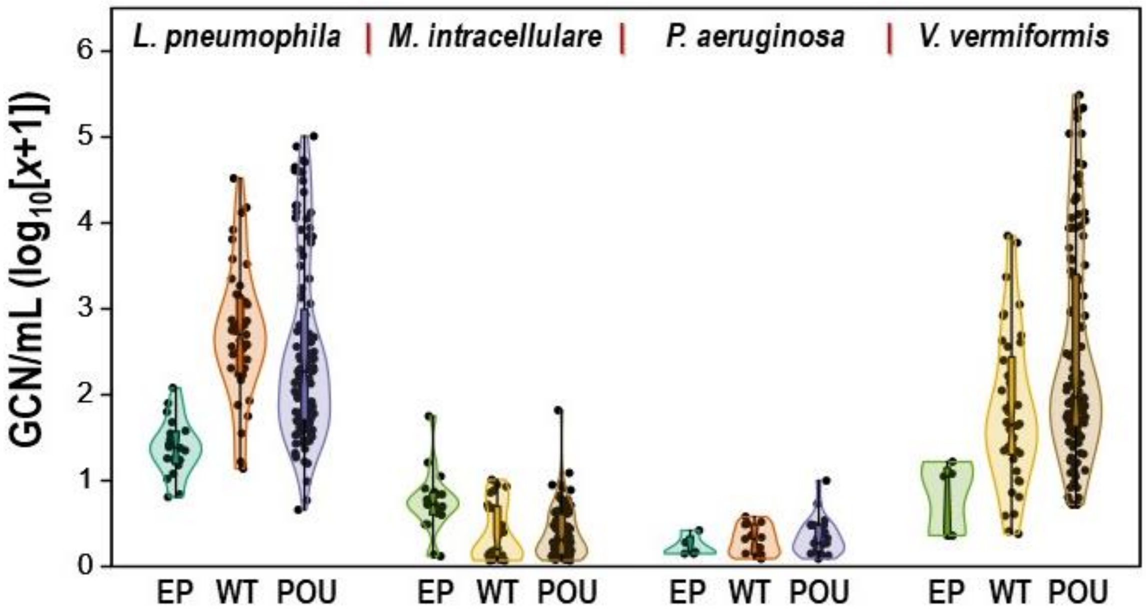

Figure S7 Cell density by quantitative polymerase chain reaction (qPCR) of selected opportunistic premise plumbing pathogens (OPPPs) and free-living amoeba (FLA) in home plumbing system (HPS) sections. Violin plot of gene copy number ($\log_{10}[x+1]$/mL) detected for *L. pneumophila*, *M. intracellulare*, *P. aeruginosa*, and *V. vermiformis* at each section and flushing event. Each dot represents a single sample event. Sections: water tank and recirculation (WT); point-of-use (POU). GCN: gene copy number.

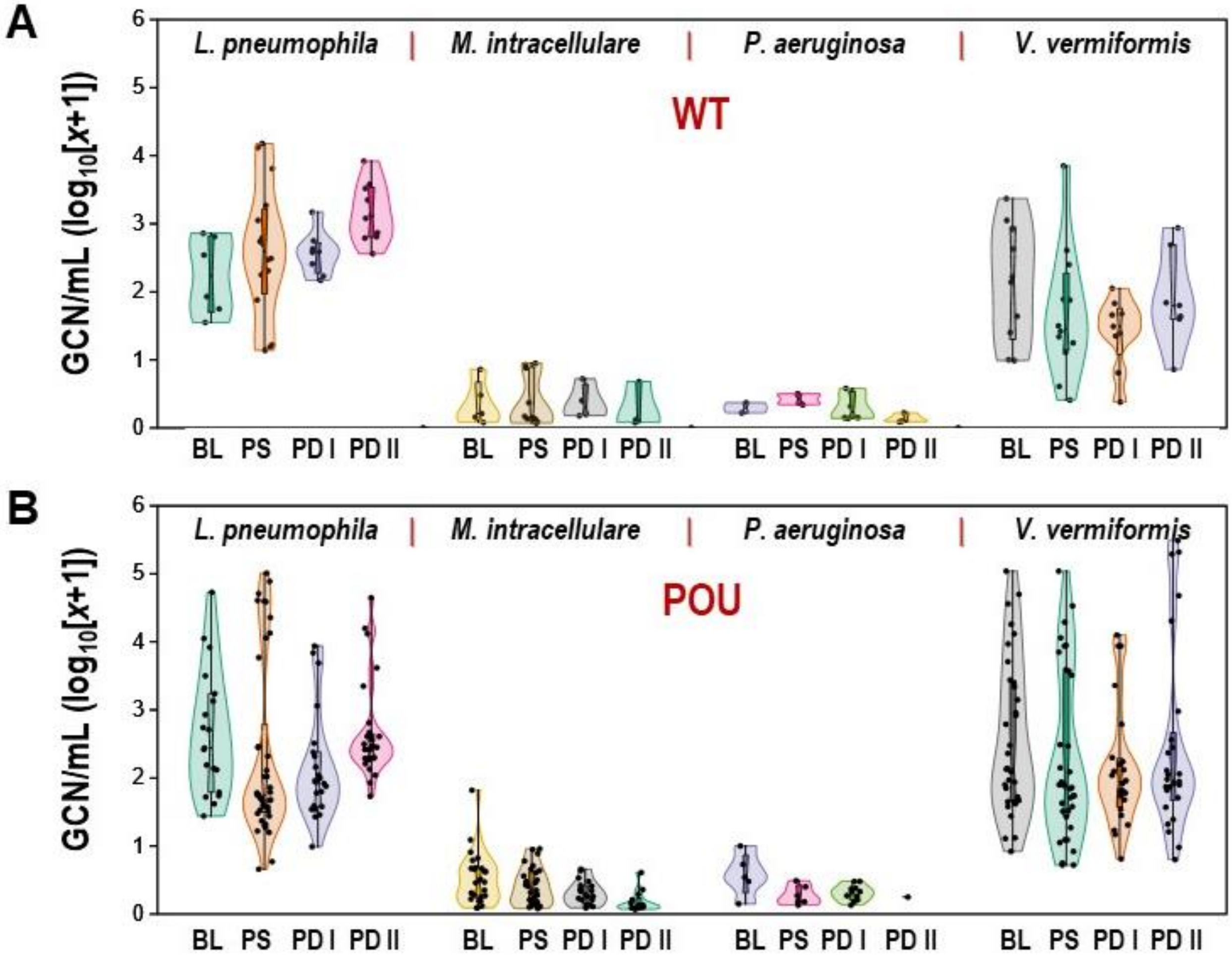

Figure S8 Cell density by quantitative polymerase chain reaction (qPCR) of selected opportunistic premise plumbing pathogens (OPPPs) and free-living amoeba (FLA) in hot water samples. Violin plot of gene copy number ($\log_{10}[x+1]$/mL) detected for *L. pneumophila*, *M. intracellulare*, *P. aeruginosa*, and *V. vermiformis* at each flushing event. Each dot represents a single sample event. Sections: water tank and recirculation (WT); point-of-use (POU). Flushing events: baseline (BL); post stagnation (PS); post drain I (PD I); post drain II (PD II). GCN: gene copy number.

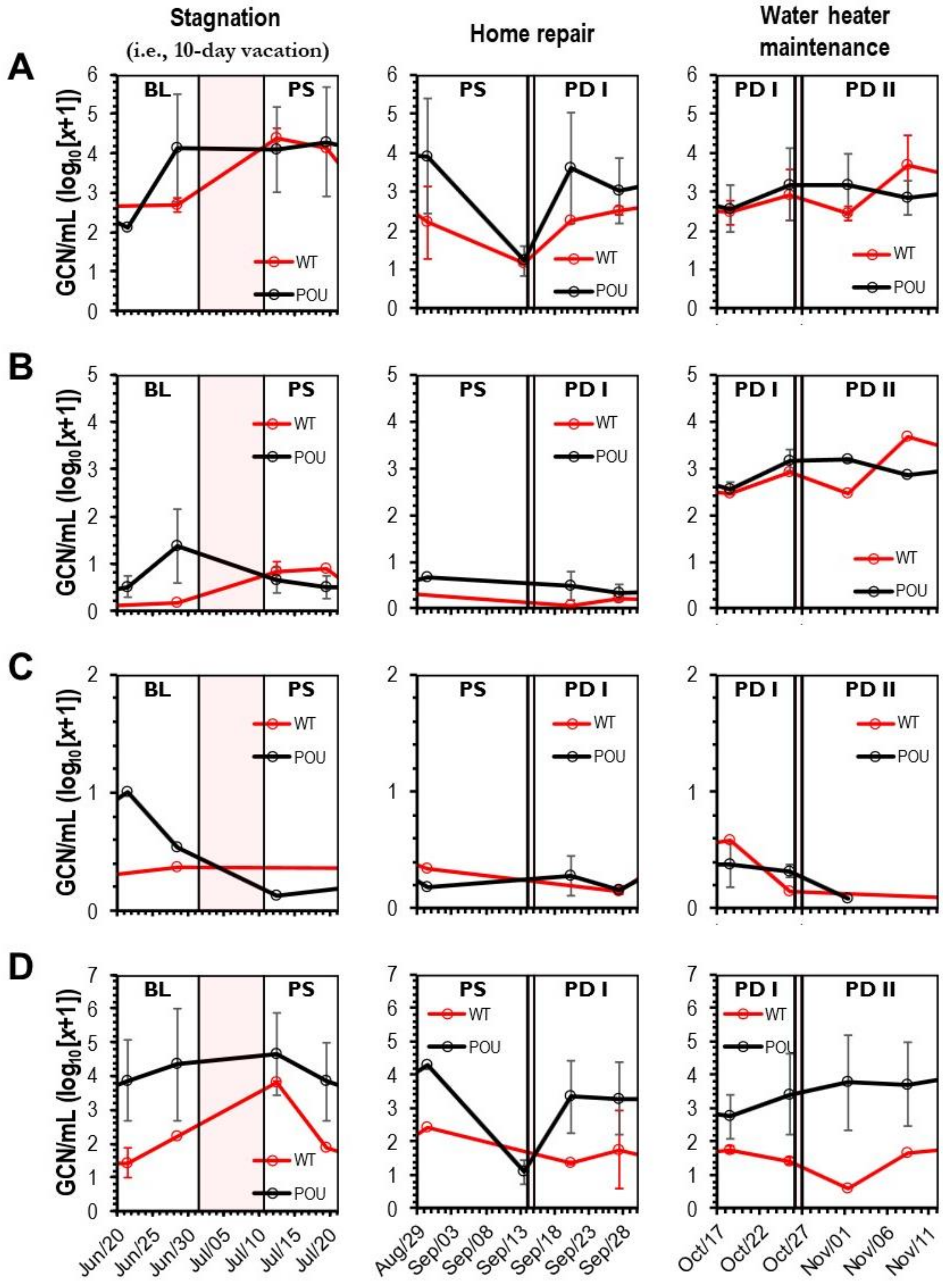

Figure S9 Effect of simulated residential activity on selected opportunistic premise plumbing pathogens (OPPPs) and free-living amoeba (FLA). Gene copy number ($\log_{10}[x+1]$/mL) by qPCR for (A) *L. pneumophila*, (B) *M. intracellulare*, (C) *P. aeruginosa*, and (D) *V. vermiformis* before and after residential activity. Sections: water tank and recirculation (WT); point-of-use (POU). Flushing events: baseline (BL); post stagnation (PS); post drain I (PD I); post drain II (PD II). GCN: gene copy number. Bars represent ±SD.

REFERENCES

Anuj, S.N., Whiley, D.M., Kidd, T.J., Bell, S.C., Wainwright, C.E., Nissen, M.D., and Sloots, T.P. (2009). Identification of *Pseudomonas aeruginosa* by a duplex real-time polymerase chain reaction assay targeting the *ecfX* and the *gyrB* genes. Diagn. Microbiol. Infect. Dis. 63:127-131. doi: 10.1016/j.diagmicrobio.2008.09.018.

Chern, E.C., King, D., Haugland, R., and Pfaller, S. (2015). Evaluation of quantitative polymerase chain reaction assays targeting *Mycobacterium avium*, *M. intracellulare*, and *M. avium* subspecies *paratuberculosis* in drinking water biofilms. J. Water Health 13:131-139. doi: 10.2166/wh.2014.060.

Donohue, M.J., O'Connell, K., Vesper, S.J., Mistry, J.H., King, D., Kostich, M., and Pfaller, S. (2014). Widespread molecular detection of *Legionella pneumophila* serogroup 1 in cold water taps across the United States. Environ. Sci. Technol. 48:3145-3152. doi: 10.1021/es4055115.

Donohue, M.J., Vesper, S., Mistry, J., and Donohue, J.M. (2019). Impact of chlorine and chloramine on the detection and quantification of *Legionella pneumophila* and *Mycobacterium* species. Appl. Environ. Microbiol. 85(24): e01942-19. doi: 10.1128/AEM.01942-19.

Kuiper, M.W., Valster, R.M., Wullings, B.A., Boonstra, H., Smidt, H., and Van Der Kooij, D. (2006). Quantitative detection of the free-living amoeba *Hartmannella vermiformis* in surface water by using real-time PCR. Appl. Environ. Microbiol. 72:5750-5756. doi: 10.1128/AEM.00085-06.

Lu, J., Struewing, I., Yelton, S., and Ashbolt, N. (2015). Molecular survey of occurrence and quantity of *Legionella* spp., *Mycobacterium* spp., *Pseudomonas aeruginosa* and amoeba hosts in municipal drinking water storage tank sediments. J. Appl. Microbiol. 119:278-288. doi: 10.1111/jam.12831.

Lytle, D.A., Formal, C., Cahalan, K., Muhlen, C., and Triantafyllidou, S. (2021). The impact of sampling approach and daily water usage on lead levels measured at the tap. Water Res. 197:117071. doi: 10.1016/j.watres.2021.117071.

Riviere, D., Szczebara, F.M., Berjeaud, J.M., Frere, J., and Hechard, Y. (2006). Development of a real-time PCR assay for quantification of *Acanthamoeba* trophozoites and cysts. J. Microbiol. Methods. 64:78-83. doi: 10.1016/j.mimet.2005.04.008.

Ryu, H., Henson, M., Elk, M., Toledo-Hernandez, C., Griffith, J., Blackwood, D., Noble, R., Gourmelon, M., Glassmeyer, S., and Santo Domingo, J. (2013) Development of quantitative PCR assays targeting 16S rRNA gene of *Enterococcus* spp. and their application to the identification of *Enterococcus* species in environmental samples. Appl. Environ. Microbiol. 79:196-204. doi: 10.1128/AEM.02802-12.

Steindor, M., Nkwouano, V., Mayatepek, E., Mackenzie, C.R., Schramm, D., and Jacobsen, M. (2015). Rapid detection and immune characterization of *Mycobacterium abscessus* infection in cystic fibrosis patients. PLoS One 10:e0119737. doi: 10.1371/journal.pone.0119737.
